# Codfish Oral Immunotherapy in Children Aged 2–10: Randomized Placebo‐Controlled Study

**DOI:** 10.1111/all.70268

**Published:** 2026-02-17

**Authors:** Agnes Sze‐yin Leung, Yanjun Gu, Ann Wing‐shan Au, Rosetta Tsz‐ching Leung, Vanessa Hiu‐tung Tang, Kin Yi Fung, Janice Min Li, Christine Yee‐yan Wai, Ting Fan Leung, Gary Wing‐kin Wong

**Affiliations:** ^1^ Department of Paediatrics, Faculty of Medicine The Chinese University of Hong Kong Hong Kong China; ^2^ Hong Kong Hub of Paediatric Excellence (HOPE) The Chinese University of Hong Kong Hong Kong China

**Keywords:** allergen immunotherapy, codfish allergy, desensitization therapy, oral immunotherapy, randomized controlled trial

## Abstract

**Background:**

For young children with fish allergy, dietary avoidance is the current standard of care. We aimed to assess whether codfish oral immunotherapy (OIT) can induce desensitization or sustained unresponsiveness (SU) in this population.

**Methods:**

We conducted a randomized, double‐blind, placebo‐controlled study in Hong Kong. Children aged 2–10 years reactive to codfish protein during DBPCFC were randomly assigned 1:1 to codfish OIT or placebo for 52 weeks (1000 mg daily maintenance) followed by 8 weeks avoidance. Primary outcome was desensitization at week 52; sustained unresponsiveness (SU) at week 60 was a key secondary outcome.

**Results:**

Between November 2022–September 2023, 70 children (median age 5.6 years) were randomized to codfish OIT (*n* = 35) or placebo (*n* = 35). At the week 52 end‐of‐treatment challenges, 15 (43%) OIT participants versus 4 (11%) placebo achieved desensitization (risk difference 32%, 95% CI 9%–51%; *p* = 0.003), with significantly greater increases in cumulative tolerated protein [median 4330 mg vs. 0 mg, *p* < 0.001]. At week 60, SU rates were 8 (23%) versus 3 (9%) (risk difference 14%, 95% CI −5% to 33%; *p* = 0.332). Codfish OIT significantly decreased codfish‐specific IgE, rGad c1‐specific IgE, and skin reactivity while increasing codfish‐specific IgG4. The treatment demonstrated acceptable safety with minimal moderate‐to‐severe adverse events and epinephrine use (3% both groups), and immunological evidence of reduced cross‐reactivity to salmon and catfish allergens.

**Conclusions:**

In children with fish allergy, codfish OIT significantly increased desensitization rates compared to avoidance, though SU remained limited. The treatment demonstrated acceptable safety with predominantly mild reactions and reduced IgE cross‐reactivity to other fish allergens.

**Trial Registration:**

ClinicalTrials.gov identifier: NCT05590299

## Graphical abstract

Codfish OIT significantly increased desensitization in children with fish allergy (43% vs. 11% placebo, *p* = 0.003), with 69% achieving 10‐fold threshold increases versus 26% in placebo (*p* = 0.0003). Sustained unresponsiveness remained limited (23% vs. 9%, *p* = 0.332). Treatment demonstrated acceptable safety with predominantly mild reactions and reduced IgE cross‐reactivity to other fish species. DBPCFC, double‐blind placebo‐controlled food challenge; DS, desensitized; FOIT, fish oral immunotherapy; Ig, immunoglobulin; ns, not significant; OIT, oral immunotherapy; SPT, skin prick test; SU, sustained unresponsiveness.

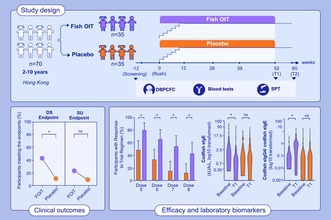

**Abbreviations**
AEs: adverse events
CI: confidence intervals
DBPCFCs: double‐blind placebo‐controlled food challenges
FEV1: forced expiratory volume in 1 s
FOIT: fish oral immunotherapy
ITT: intention‐to‐treat
MeHg: methylmercury
NNT: number needed to treat
OIT: oral immunotherapy
PWH: Prince of Wales Hospital
RCT: randomized controlled trial
SAEs: serious adverse events
sIgE: specific IgE
SPT: skin prick test
SU: sustained unresponsiveness
TEAEs: treatment‐emergent adverse events

## 1 Introduction

Fish allergy represents a persistent and potentially life‐threatening condition that predominantly affects children in coastal regions where fish constitutes a dietary staple [1]. Unlike milk and egg allergies that often resolve naturally (80% by late adolescence) [2, 3], fish allergy demonstrates a high degree of persistence, with only one in five affected children achieving resolution within 2–5 years post‐diagnosis [4], creating a lifelong burden of strict avoidance, emergency preparedness, and social limitations [5]. Current management relies solely on allergen avoidance and emergency treatment, yet significant gaps persist in patient preparedness and adherence to safety protocols [6]. With no curative treatments available and mounting evidence supporting the efficacy of oral immunotherapy (OIT) for other persistent food allergies [7], there exists an urgent need for systematic investigation of fish OIT as a potential therapeutic intervention.

Consequently, we developed the first randomized, blinded, placebo‐controlled trial of codfish OIT in children aged 2–10 years to test the hypothesis that consumption of escalating subthreshold amounts of fish enhances immune tolerance and provides a safe, tolerable treatment option for children with persistent fish allergies. In this randomized controlled trial (RCT), participants underwent a 52‐week blinded treatment period with either codfish OIT or placebo OIT. We employed double‐blind placebo‐controlled food challenges (DBPCFCs) to evaluate desensitization following the 52‐week treatment period and to assess sustained unresponsiveness (SU) after the 8‐week no‐exposure period.

## 2 Methods

### 2.1 Study Design and Participants

This single‐center, randomized, double‐blind, placebo‐controlled study was conducted at the Prince of Wales Hospital (PWH) in Hong Kong. The protocol was approved by the Joint CUHK‐NTEC Clinical Research Ethics Committee (2021.666). Full description of all methods can be found in the Supporting Information Appendix.

Children aged 2–10 years weighing > 7 kg were eligible if they had confirmed codfish allergy by failed DBPCFC and positive skin prick test (SPT) (≥ 3 mm wheal with commercial codfish extract, ALK‐Abello, USA) or codfish‐specific IgE (sIgE) (≥ 0.35 kUA/L). Key exclusions included reaction to placebo during screening DBPCFC, severe anaphylaxis history (persistent hypotension, collapse, unconsciousness, hypoxia, or requiring > 3 epinephrine doses), poorly controlled asthma (FEV1 < 85% in children ≥ 8 years), beta‐blocker/ACE inhibitor use, and eosinophilic esophagitis (the full list of exclusion criteria is presented in the Supporting Information Appendix). Participants were recruited through the PWH Pediatric Department and referrals from other hospitals. Written informed consent was obtained from the parents or guardians of the participating children.

### 2.2 Randomization and Masking

Participants were randomly allocated (1:1) to codfish OIT or placebo using computerized randomization with pre‐generated sequences by an independent statistician (Figure S1). Blinding was maintained for all participants and research personnel except an unmasked site dietitian who dispensed treatments based on randomization codes from the electronic data capture system. During DBPCFCs, challenge sequences were randomly determined by an unmasked dietitian who prepared materials, while all other staff remained blinded. No inadvertent unblinding occurred throughout the study.

### 2.3 Procedure

Eligible participants underwent standardized screening including SPT, DBPCFCs, and immunological assessments (Supporting Information Appendix, 10–11). Throughout this study, codfish ingestion is defined in mg of codfish protein unless otherwise specified.

Following randomization, participants received either codfish OIT or matched placebo for daily administration. The active treatment consisted of Atlantic codfish ( *Gadus morhua* ) blended in potato, corn, carrot, onion, herbs and spices, while placebo contained identical ingredients excluding codfish. Both products maintained similar appearance and taste, with consistent preparation methods used for treatment products and DBPCFC materials (investigational product [IP] details are presented in the Supporting Information Appendix, Section II).

The immunotherapy protocol comprised three phases: rush induction (day 1) with 20‐min dose escalations from 3.7 mg to maximum 120 mg codfish protein (Table S1); buildup phase over 12 weeks with biweekly increases from 180 mg to target maintenance of 1000 mg daily (Table S2); and maintenance phase continuing 1000 mg daily for the remaining 52‐week period. Dose modifications followed pre‐established protocols for reactions and illness (Table S3). Participants maintained strict dietary fish avoidance throughout the study, with adherence monitored through daily diaries and product accountability records.

DBPCFCs were conducted at study entry, week 52, and week 60 (only for those who passed the week 52 challenge) using eight escalating codfish protein doses. For ages 2–6 years, doses progressed from 10 mg to 5000 mg (total 13,330 mg, equivalent to 80 g fish); for ages 7–10 years, from 30 mg to 7333 mg (total 17,663 mg, equivalent to 106 g fish) (Tables S4–S6, Supporting Information Appendix, 23–24). Participants completing the week 52 DBPCFC were classified as: (1) fully desensitized if they passed the full‐dose challenge at week 52; (2) partially desensitized if they failed the week 52 challenge but tolerated a higher cumulative dose than at the screening challenge; (3) persistently allergic if their reaction threshold remained unchanged from baseline or decreased at week 52; or (4) achieved SU if they passed the full‐dose challenge at week 60 after 8 weeks of avoidance.

Immunological evaluations at screening, treatment end (T1), and 8 weeks post‐treatment (T2) included SPT using standardized extracts (codfish, salmon, catfish and fish mix consisting of codfish, halibut and flounder), with histamine as positive control and saline as negative control. Blood samples measured total IgE, codfish, catfish, salmon and rGad c 1‐specific IgE, and codfish‐specific IgG4 levels by ImmunoCAP (Phadia AB, Uppsala, Sweden). Basophil activation was assessed by measuring CD63 expression upregulation using flow cytometry analysis.

### 2.4 Outcomes

The primary endpoint was full desensitization at T1, defined as successfully completing the DBPCFC with absence of reaction to 13 g codfish protein (80 g fish) for ages 2–6 years or 17 g codfish protein (106 g fish) for ages 7–10 years.

Secondary outcomes included SU (passing both T1 and T2 DBPCFCs after 8 weeks off treatment), maximum tolerated dose at T1, changes in codfish SPT wheal diameter and serum specific IgE/IgG4 levels, in addition to safety parameters including exposure‐adjusted adverse event rates, blood methylmercury and urine microplastic concentrations.

Safety monitoring involved adverse event documentation from study initiation through T1, with participants maintaining daily diaries reviewed at each visit. Investigators assessed causality as unrelated, unlikely, possibly, or probably treatment‐related. Allergic reactions were graded using NIH NIAID Consortium criteria [8], non‐allergic events per ICH guidelines. Severe adverse events interfered with daily functioning requiring therapeutic management, while serious adverse events followed established standard definitions.

### 2.5 Statistical Analysis

Sample size was calculated using a two‐sample *χ* ^2^ test at 0.05 significance level, assuming 15% placebo and 50% codfish OIT desensitization rates with 30% dropout. To achieve 80% power, 62 participants were required; accounting for attrition, 70 participants were enrolled (35 per group).

Statistical methodology followed established protocols for RCTs, with intention‐to‐treat (ITT) analysis as the primary approach. The per‐protocol population for desensitization and SU was defined as all ITT participants who adhered to maintenance dosing protocols and had evaluable DBPCFC assessments at T1 and T2 timepoints. Primary assessments were conducted in the ITT population unless otherwise specified, with immunological analyses performed in the per‐protocol sample.

The study was deemed successful if primary efficacy analysis achieved statistical significance (*p* < 0.05) with superior full desensitization rates in the OIT group compared to placebo. Treatment differences in desensitization percentages (OIT minus placebo) were estimated with 95% CIs. Secondary efficacy analyses for SU followed identical methodology. Descriptive statistics for demographic and baseline characteristics were presented as means with standard deviations (or medians with interquartile ranges for non‐normal distributions), while categorical variables were summarized as frequencies and percentages. Between‐group differences in continuous outcomes (including wheal size, immunoglobulin levels) were analyzed using the Wilcoxon rank‐sum test. Inter‐subgroup differences at the same timepoints will be analyzed using Kruskal–Wallis tests for overall group comparisons, with pairwise Wilcoxon rank‐sum tests and Bonferroni correction for post hoc analyses. Longitudinal measures, including basophil activation and immunoglobulin levels, were analyzed using repeated‐measures models, adjusting for baseline values. Statistical testing employed a 5% significance threshold (*α* = 0.05) with 95% confidence intervals (CI) reported throughout.

Statistical computations were performed using IBM SPSS Statistics (Version 29.0.1.0) and GraphPad Prism version 10.2.3 for Mac. The trial was prospectively registered with ClinicalTrials.gov prior to participant enrolment (NCT05590299).

## 3 Results

**FIGURE 1:**
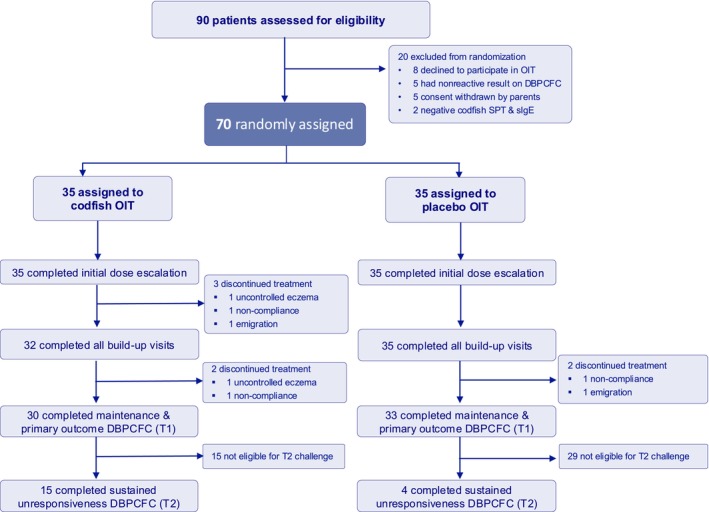
The trial profile with the CONSORT flow diagram. DBPCFC, double‐blind placebo‐controlled food challenge; OIT, oral immunotherapy.

Between November 2022 and September 2023, 90 individuals were screened and 70 participants randomized equally (35 per group) (Figure 1). All participants were Chinese, with a male predominance of 63%, and a mean age of 5.6 years (SD 2.5), including 70% aged 2–6 years and 30% aged 7–10 years (Table 1). Both groups had comparable but below‐average weight and height z‐scores (OIT: −0.78, −0.67; Placebo: −0.89, −0.75). Similar proportions had fish‐induced anaphylaxis history (11% vs. 9%) and atopic dermatitis (60% vs. 69%). The only notable baseline difference was a higher prevalence of other food allergies in the OIT group (74% vs. 46%) (Table S8C). Groups showed similar baseline fish reactivity, with median eliciting doses of 300 mg versus 900 mg, and overlapping interquartile ranges. Key allergy biomarkers including SPT wheal size, codfish‐specific IgE, Gad c 1‐specific IgE, basophil activation response, and total IgE were comparable between groups.

**TABLE 1:** Demographics and baseline characteristics.

	FOIT (*n* = 35)	Placebo (*n* = 35)	Total
Sex: male, *n* (%)	22 (62.9)	22 (62.9)	44 (62.9)
Age (y), mean (SD)	5.5 (2.5)	5.8 (2.6)	5.6 (2.5)
2–6 years, *n* (%)	25 (71.4)	24 (68.6)	49 (70.0)
7–10 years, *n* (%)	10 (28.6)	11 (31.4)	21 (30.0)
Weight, median (IQR), kg	15.1 (4.7)	15.1 (6.6)	17.2 (8)
Weight *z*‐scores, median (IQR)	−0.78 (1.23)	−0.89 (1.78)	−0.62 (1.59)
Height, median (IQR), cm	98 (18)	103 (21)	109 (28)
Height, *z*‐scores, median (IQR)	−0.67 (1.62)	−0.75 (1.27)	−0.61 (1.26)
Weight for height *z*‐scores, median (IQR)	−0.42 (1.11)	−0.41 (1.85)	−0.42 (1.43)
BMI *z*‐scores, median (IQR)	−0.37 (1.17)	−0.59 (1.88)	−0.39 (1.68)
Allergic disease at baseline, *n* (%)			
Age of fish allergy onset (months), median (IQR)	9 (8, 12)	9 (7, 12)	9 (7, 12)
History of fish‐induced anaphylaxis, *n* (%)^a^	4 (11.4)	3 (8.6)	7 (10.0)
OFC‐confirmed other fish allergies, *n* (%)			
Salmon	8/12 (66.7)	9/16 (56.3)	17/28 (60.7)
Grass carp	6/6 (100)	11/11 (100)	17/17 (100)
Atopic dermatitis	21 (60.0)	24 (68.6)	45 (64.3)
Asthma	1 (2.9)	3 (8.6)	4 (5.7)
Food allergies other than fish	26 (74.3)	16 (45.7)	42 (60.0)
Baseline reactivity to fish, median (IQR)			
Eliciting dese, mg codfish protein	300 (90, 3000)	900 (90, 900)	300 (90, 1425)
Cumulative tolerated dose, mg codfish	130 (10, 1330)	430 (40, 430)	130 (40, 430)
SPT MWD to codfish (mm)	5.0 (3.5, 6.5)	4.5 (3.5, 5.5)	4.5 (3.5, 5.6)
Codfish‐specific IgE (kUA/L)	4.2 (1.4, 8.3)	3.0 (0.9, 6.3)	3.3 (1.1, 8.3)
rGad c1‐specific IgE (kUA/L)	11.4 (5.0, 26.3)	13.1 (4.5, 34.6)	11.4 (4.5, 27.0)
BAT to codfish (CD63%)	66.4 (36.3, 85.6)	68.1 (7.3, 79.5)	66.4 (19.8, 83.0)
Total IgE (kUA/L)	66.4 (36.3, 85.6)	68.1 (7.3, 79.5)	66.4 (19.8, 83.0)

Abbreviations: IQR, interquartile range; MWD, mean wheal diameter; SD, standard deviation; y, year.

^a^ The fish species resulting in anaphylaxis varied among participants and included grass carp (*n* = 1), salmon (*n* = 1), yellow sea bream (*n* = 1), grouper (*n* = 1), and other unspecified fish species (*n* = 3).

All participants completed the initial dose escalation phase, constituting the ITT population (*n* = 70). During the buildup phase, three participants in the active group discontinued due to uncontrolled eczema, non‐compliance, and emigration, resulting in 32 active and 35 placebo participants completing this phase (Table S16). For the primary outcome assessment (T1 DBPCFC), 30 active and 33 placebo participants completed the evaluation (*n* = 63). For the secondary outcome (T2 DBPCFC) intended for those who passed T1, 15 active and 4 placebo participants completed the assessment (*n* = 19).

Protocol adherence patterns remained consistent throughout both treatment phases (Table S13A). The total dose administration (taken) averaged 330.8 (SD 106.4) doses per participant in the active group versus 354.4 (SD 61.5) in the placebo group, with participants completing an average of 89% of prescribed doses across both study arms. During the buildup phase, the median percentage of missed doses was 7.5% (IQR 1.5%–14.0%) for active treatment and 6.0% (IQR 0.0%–13.5%) for placebo. During the maintenance phase, missed dose rates were comparable at 6.5% (IQR 4.0–16.0) for active treatment and 6.0% (IQR 1.0–16.0) for placebo. Episodes of five or more consecutive missed doses in both groups were primarily attributed to concurrent illnesses during the buildup phase (60% FOIT, 50% placebo). During the maintenance phase, the main reason shifted to travel (72% FOIT, 41% placebo). During buildup, these interruptions affected 10 (29%) participants in the active group and 11 (31%) in the placebo group (Table S14). During maintenance, the frequency increased substantially but was comparable between treatment groups (13 [41%] in active and 15 [43%] in placebo groups) (Table S15). No significant differences were observed between participants with fewer than 5 versus 5 or more missed doses in baseline and outcome parameters (Table S13B).

### 3.1 Clinical Outcomes

**FIGURE 2:**
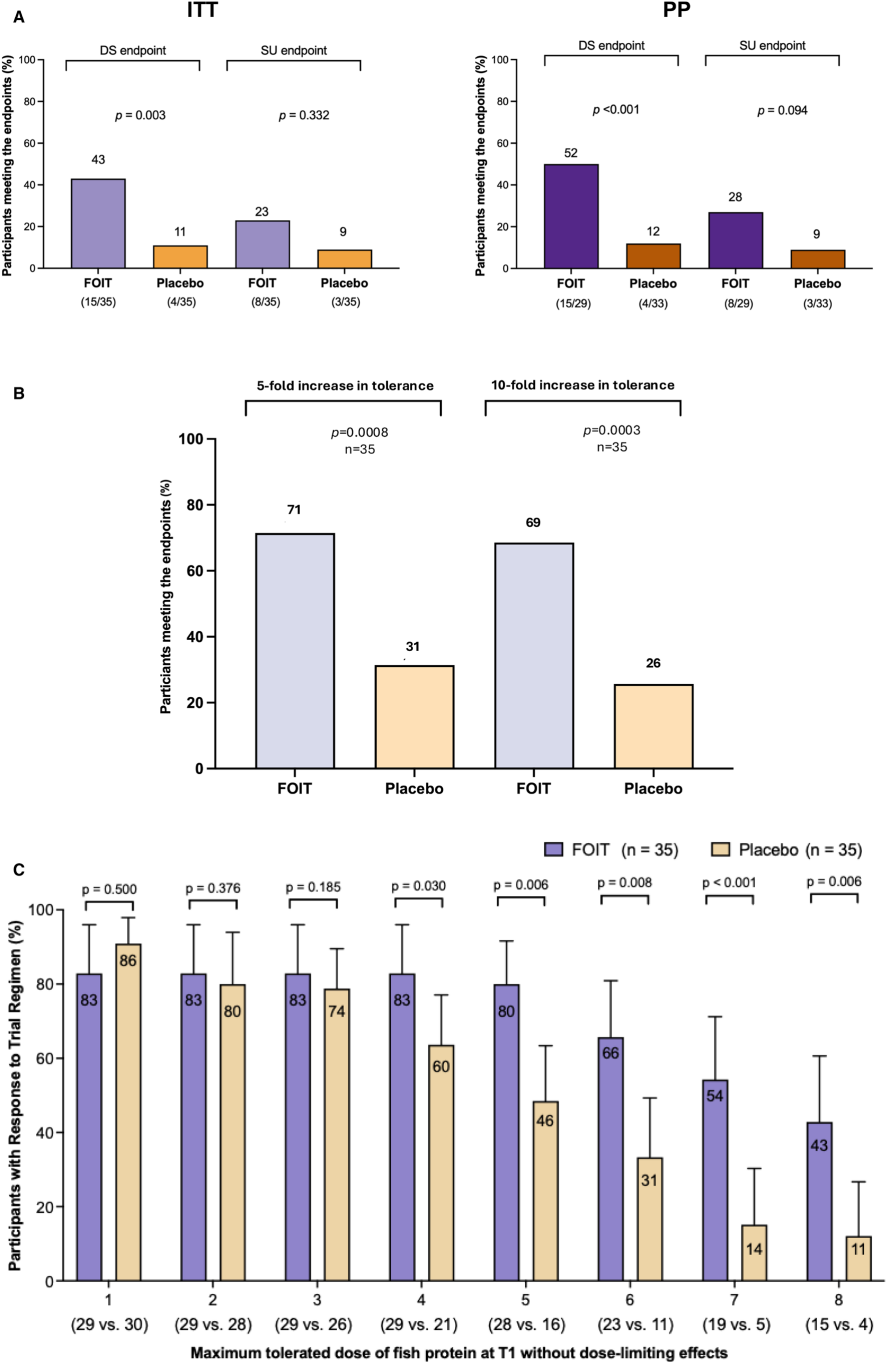
The primary and secondary outcomes. (A) Data are shown for the primary endpoint (desensitization) at week 52 and secondary endpoint (sustained unresponsiveness) at week 60, measured by DBPCFC for the ITT sample and per‐protocol (PP) sample, comparing codfish oral immunotherapy and placebo groups. (B) Data showing a fold change increase in tolerated codfish protein dose between baseline and T1. A 5‐fold increase means T1 tolerated protein/baseline tolerated protein ≥ 5. A 10‐fold increase means T1 tolerated protein/baseline tolerated protein ≥ 10. (C) Data displaying the cumulative percentage of participants who successfully reached each dose level without dose‐limiting effects at week 52 (T1 challenge). For ages 2–6 years, the dose progression was as follows: 10 mg → 30 mg → 90 mg → 300 mg → 900 mg → 3000 mg → 4000 mg → 5000 mg (Total cumulative dose: 13,330 mg, equivalent to 80 g fish); for ages 7–10 years, the dose progression was as follows: 30 mg → 90 mg → 300 mg → 900 mg → 3000 mg → 6000 mg → 7333 mg (Total cumulative dose: 17,663 mg, equivalent to 106 g fish).

At week 52, codfish OIT resulted in significantly higher rates of full desensitization compared to placebo (Table 2). The ITT analysis showed a desensitization rate of 43% (95% CI 26%–60%) in the codfish OIT versus 11% (95% CI 0%–23%) in the placebo group, corresponding to a statistically significant absolute risk difference of 32% (95% CI 9%–51%; *p* = 0.003) (Figure 2A). This clinical benefit was supported by a risk ratio of 4.13 (95% CI 1.54 to 11.07) and a corresponding number needed to treat (NNT) of 4 (95% CI 2–12). In the per‐protocol population, desensitization rates were 52% (95% CI 32%–71%) for active treatment versus 12% (95% CI 0%–24%) for placebo, yielding a difference of 40% (95% CI 14%–60%; *p* < 0.001). This result was reinforced by multiple sensitivity analyses (Sensitivity 2, 3, and adjusted models in Table S8A and Figure S5), which consistently showed significant risk differences ranging from 26% to 40% (*p*‐values from < 0.001 to 0.036).

**TABLE 2:** Treatment efficacy outcomes: desensitization and sustained unresponsiveness rates.

	FOIT		Placebo		Difference	*p*
	*N*	Probability (95% CI)	*N*	Probability (95% CI)	Probability (95% CI)	
Probability of desensitization						
ITT	15/35	0.43 (0.26, 0.60)	4/35	0.11 (0.00, 0.23)	0.32 (0.09, 0.51)	0.003
PP	15/29	0.52 (0.32, 0.71)	4/33	0.12 (0.00, 0.24)	0.40 (0.14, 0.60)	< 0.001
Sensitivity 1	14/35	0.40 (0.23, 0.57)	6/35	0.17 (0.04, 0.30)	0.23 (0.00, 0.43)	0.063
Sensitivity 2	14/35	0.40 (0.23, 0.57)	4/35	0.11 (0.03, 0.23)	0.29 (0.06, 0.48)	0.013
Sensitivity 3	15/35	0.43 (0.26, 0.60)	6/35	0.17 (0.04, 0.30)	0.26 (0.02, 0.46)	0.036
Probability of sustained unresponsiveness						
ITT	8/35	0.23 (0.10, 0.41)	3/35	0.09 (−0.01, 0.19)	0.14 (−0.05, 0.33)	0.332
PP	8/29	0.28 (0.10, 0.45)	3/33	0.09 (−0.01, 0.19)	0.19 (−0.03, 0.40)	0.094
Sensitivity 1	7/35	0.20 (0.06, 0.34)	5/35	0.14 (0.02, 0.26)	0.06 (−0.14, 0.25)	0.752
Sensitivity 2	7/35	0.20 (0.06, 0.34)	3/35	0.09 (−0.01, 0.18)	0.11 (−0.08, 0.30)	0.306
Sensitivity 3	8/35	0.23 (0.08, 0.37)	5/35	0.14 (0.02, 0.26)	0.09 (−0.12, 0.28)	0.540

*Note:* ITT, all participants as randomized; PP, excludes withdrawals and protocol deviations; Sensitivity 1, worst‐case for FOIT (dupilumab case = failure, placebo withdrawals = success); Sensitivity 2, all withdrawals & dupilumab = failure; Sensitivity 3: dupilumab case as observed, placebo withdrawals = success.

Sustained unresponsiveness assessed 8 weeks after stopping treatment showed more modest between‐group differences. In the ITT analysis, 23% (95% CI 10%–41%) of codfish OIT participants achieved SU compared to 9% (95% CI 0%–19%) of placebo recipients, representing a non‐significant difference of 14% (95% CI −5% to 33%; *p* = 0.332). The per‐protocol analysis showed similar non‐significant results (28% vs. 9%, difference 19% [95% CI −38% to 40%]; *p* = 0.094). Exploratory subgroup analyses suggested higher treatment effects in younger children (2–6 years: SU 14% vs. 0%, *p* = 0.048) compared to older children (7–10 years: 17% vs. 15%, *p* = 1.000), though these analyses were underpowered (Table S8B). Desensitization and SU rates were similar between participants with ≥ 5 consecutive missed doses (*n* = 41) versus those with < 5 consecutive missed doses (*n* = 29) (27% vs. 28%, *p* = 1.000; and 15% vs. 17%, *p* = 1.000, respectively) (Table S13B).

While natural threshold fluctuations occurred in the placebo group (median change 0 mg, IQR: 0, 3900 mg; 26% achieving 10‐fold increase), OIT participants demonstrated substantially greater improvement, with a median increase of 4330 mg (IQR: 390, 13,330, *p* < 0.001) and 69% achieving a 10‐fold threshold increase at week 52 (*p* = 0.0003) (Figure 2B). Challenge outcomes demonstrated dose‐dependent desensitization, with significant OIT superiority at Dose 4 (300 mg; OIT 83% vs. placebo 60%; *p* = 0.030) and sustained through Dose 8 (5000–7333 mg; OIT 43% vs. placebo 11%; *p* = 0.006) (Figure 2C).

### 3.2 Laboratory Biomarkers

**FIGURE 3:**
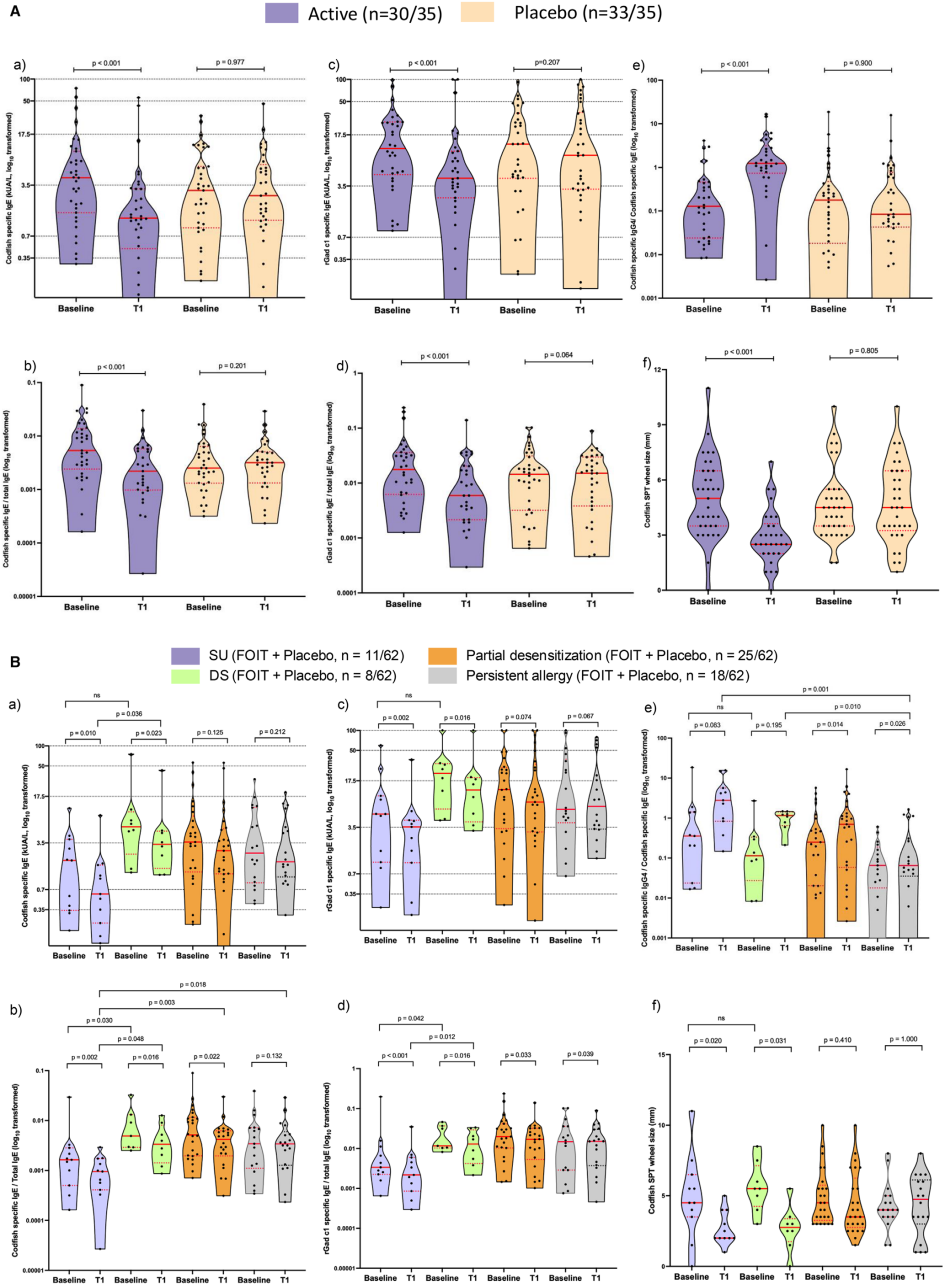
The immunological changes over the course of the study. Data are presented for the sample of per‐protocol participants at different timepoints, including before treatment and at week 52 ± week 60 of the study. Data are shown as median with interquartile range. Dashed horizontal lines indicate ImmunoCAP sIgE class cut‐off values (classes 1–5). (A) The panels show levels of codfish‐specific IgE (a), codfish‐specific IgE to total IgE ratios (b), rGad c 1‐specific IgE (c), rGad c 1 to total IgE ratio (d), codfish‐specific IgG4 to IgE ratios (e), and codfish SPT wheal diameters (f) for the codfish OIT vs. placebo treatment groups. (B) Participants receiving codfish OIT were categorized as sustained unresponsiveness (SU; *n* = 11), indicating they passed the challenge after 8 weeks of avoidance; fully desensitized (DS; *n* = 8), who passed the end‐of‐treatment challenge but reacted after avoidance; partially desensitized (*n* = 25), with increased thresholds from baseline without passing the full challenge; and persistent allergy (*n* = 18) group with no change in reactivity or decreased tolerated dose. The panels show levels of codfish‐specific IgE (a), codfish‐specific IgE to total IgE ratios (b), rGad c 1‐specific IgE (c), rGad c 1 to total IgE ratio (d), codfish‐specific IgG4 to IgE ratios (e), codfish SPT wheal diameters (f), and for the different clinical outcome groups.

Longitudinal assessment of immune parameters revealed distinct patterns between treatment groups (Figure 3A and Table S9) and according to treatment outcomes (Figure 3B and Table S10). Relative to placebo, codfish OIT produced significant reductions in codfish‐specific IgE, rGad c 1‐specific IgE, skin prick test wheal diameters (all *p* < 0.001), and basophil activation responses (*p* = 0.016) (Figure S6), while simultaneously promoting increases in codfish‐specific IgG4 levels at week 52 (*p* < 0.001).

Analysis of immune parameters was stratified by treatment response. Participants achieving SU (*n* = 11) showed the lowest codfish and rGad c 1‐specific IgE to total IgE ratio both at the start and after treatment, but not with SPT wheal diameters and codfish‐specific IgG4 levels (Figure 3B). Although baseline codfish‐specific IgE levels did not differ significantly across all response groups (Kruskal‐Wallis test), exploratory *post hoc* pairwise analysis with Bonferroni correction suggested lower levels in the SU versus DS subgroups (*p* = 0.048). After 8‐week avoidance (T2), codfish‐specific IgG4 levels remained stable in the SU group but decreased in the desensitization group (*p* = 0.048) (Figure S7).

**FIGURE 4:**
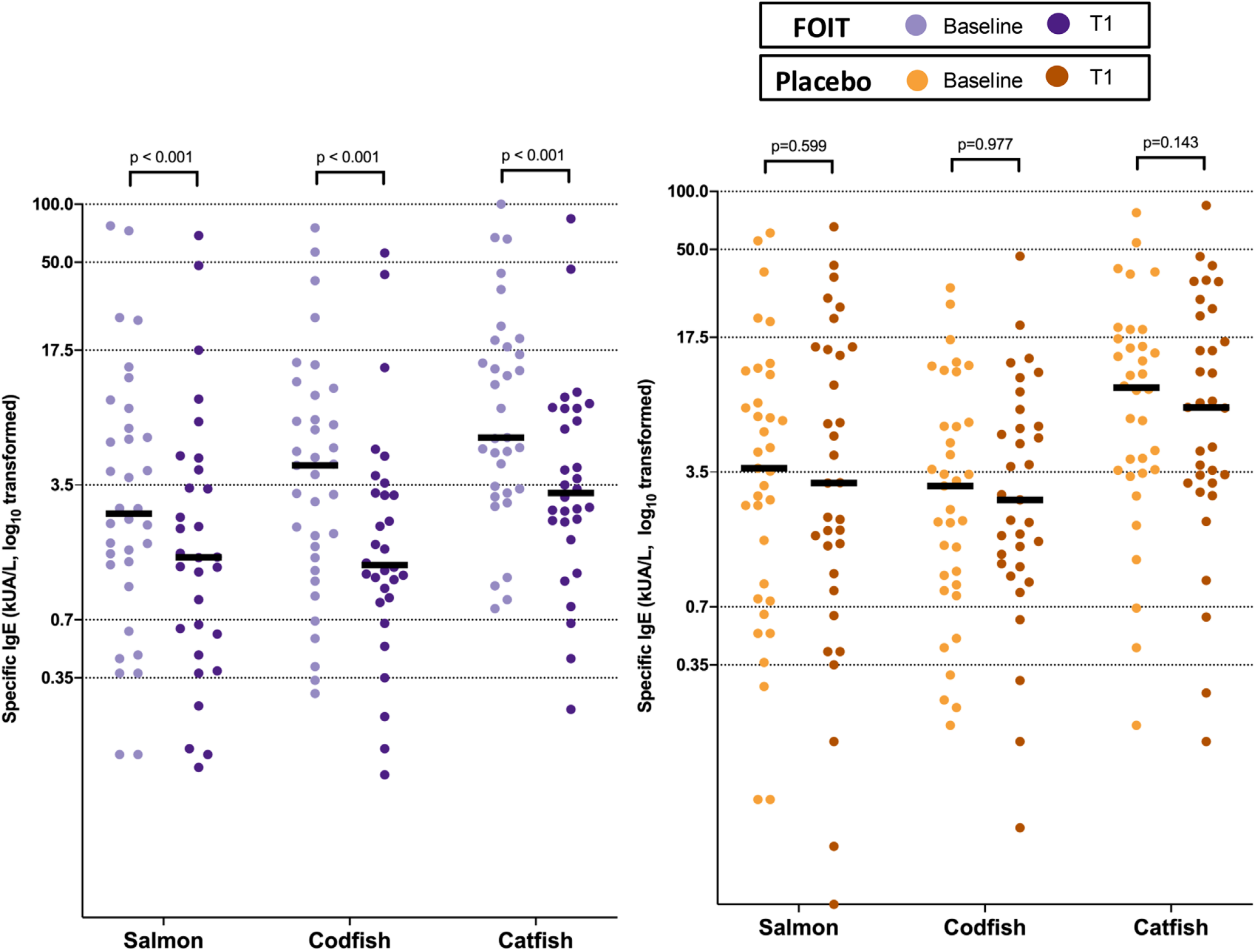
The change in salmon, codfish, and catfish‐specific IgE levels from baseline to T1 between codfish OIT vs. placebo treatment groups. Dashed horizontal lines indicate ImmunoCAP sIgE class cut‐off values (classes 1–5). Median levels are shown.

Codfish OIT also showed an immunological cross‐desensitization effect. Codfish OIT not only significantly reduced allergen‐specific IgE levels to codfish (4.4 kUA/L [IQR 1.5–10.2] at baseline to 1.2 kUA/L [IQR 0.4–3.1] at T1, *p* < 0.001) but also decreased IgE levels to salmon and catfish. At week 52, salmon‐specific IgE dropped from 2.5 kUA/L (IQR 1.3–7.3) to 1.5 kUA/L (IQR 0.6–4.3) (*p* < 0.001), and catfish‐specific IgE decreased from 6.2 kUA/L (IQR 3.3–19.0) to 3.2 kUA/L (IQR 2.1–8.7) (*p* < 0.001) (Figure 4). This suggests that monotherapy with codfish may provide clinical protection against a wider range of fish species.

### 3.3 Adverse Reactions

Dosing reactions occurred in 80% of OIT participants versus 43% receiving placebo, most frequently during build‐up phase (66% vs. 40%) (Table S12). Treatment‐related moderate and severe treatment‐emergent adverse events (TEAEs) were observed only in the OIT group (9% and 3%, respectively) (Table S11B). One participant per group (3%) required epinephrine: the OIT participant for a treatment‐related reaction during buildup, and the placebo participant during exit challenge. The same OIT participant had an additional epinephrine‐requiring wheat anaphylaxis from accidental exposure.

Most participants experienced TEAEs unrelated to treatment (86% FOIT, 94% placebo), primarily acute infections (74% FOIT, 86% placebo). Accidental allergen exposures were similar between groups (57% FOIT, 66% placebo). Baseline food challenge‐related TEAEs were comparable (77% FOIT, 86% placebo) (Table S11A). No treatment‐related SAEs occurred.

Codfish OIT participants experienced allergic reactions at 4.5 times the rate of placebo (IRR = 4.50, *p* < 0.0001) (Table 3). Older children (7–10 years) had higher reaction rates than younger children (IRR = 48.37 vs. 3.37), driven primarily by cutaneous reactions (IRR = 35.18 vs. 3.18, both *p* < 0.0001). Conversely, gastrointestinal symptoms, particularly vomiting, showed an 8.6‐fold increase (IRR = 8.48, *p* = 0.0173) in younger children. Respiratory symptoms overall did not differ significantly between groups (IRR = 1.64, *p* = 0.1134). Severe respiratory events remained uncommon with no significant differences between groups.

**TABLE 3:** Exposure‐adjusted incidence rates of treatment‐related adverse events during the intervention period.

	Exposure‐adjusted incidence^a^		FOIT versus placebo		
	FOIT (*n* = 35)	Placebo (*n* = 35)	IRR^b^	95% CI	*p*
*Overall summary*					
2–6 years	5.58	1.66	3.37	2.33–4.96	< 0.0001
7–10 years	4.64	0.10	48.37	8.24–1953.20	< 0.0001
All age groups	5.30	1.18	4.50	3.17–6.52	< 0.0001
*Cutaneous symptoms*					
2–6 years	4.46	1.40	3.18	2.12–4.87	< 0.0001
7–10 years	3.37	0.10	35.18	5.88–1432.20	< 0.0001
All age groups	4.13	1.00	4.13	2.81–6.21	< 0.0001
*Localized urticaria*					
2–6 years	3.78	0.77	4.94	2.95–8.74	< 0.0001
7–10 years	1.05	0.10	10.99	1.56–477.04	0.004
All age groups	2.97	0.56	5.30	3.21–9.19	< 0.0001
*Generalized urticaria*					
2–6 years	0.23	0.00	—	—	—
7–10 years	0.11	0.00	—	—	—
All age groups	0.19	0.00	—	—	—
*Cutaneous angioedema*					
2–6 years	0.72	0.09	8.48	1.99–75.98	0.0005
7–10 years	2.32	0.00	—	—	—
All age groups	1.20	0.06	20.36	5.26–174.17	< 0.0001
*Erythema*					
2–6 years	0.27	0.68	0.40	0.13–1.07	0.0477
7–10 years	0.32	0.00	—	—	—
All age groups	0.28	0.47	0.60	0.23–1.45	0.2273
*Gastrointestinal symptoms*					
2–6 years	0.90	0.30	3.03	1.23–8.47	< 0.0001
7–10 years	0.11	0.00	—	—	—
All age groups	0.66	0.21	3.21	1.32–8.95	0.0047
*Vomiting*					
2–6 years	0.36	0.04	8.48	1.14–376.08	0.0173
7–10 years	0.00	0.00	—	—	—
All age groups	0.25	0.03	8.57	1.15–380.31	0.0166
*Diarrhea*					
2–6 years	0.27	0.17	1.59	0.38–7.66	0.4912
7–10 years	0.00	0.00	—	—	—
All age groups	0.19	0.12	1.61	0.38–7.74	0.4804
*Abdominal pain*					
2–6 years	0.41	0.09	4.77	0.99–45.34	0.0308
7–10 years	0.11	0.00	—	—	—
All age groups	0.32	0.06	5.36	1.14–50.28	0.0166
*Respiratory symptoms*					
2–6 years	0.63	0.72	0.87	0.40–1.88	0.7113
7–10 years	1.26	0.00	—	—	—
All age groups	0.82	0.50	1.64	0.86–3.22	0.1134
*Rhinorrhea/sneezing*					
2–6 years	0.18	0.72	0.25	0.06–0.76	0.0064
7–10 years	0.11	0.00	—	—	—
All age groups	0.16	0.50	0.32	0.09–0.89	0.0164
*Coughing*					
2–6 years	0.32	0.09	3.71	0.71–36.58	0.0923
7–10 years	0.11	0.00	—	—	—
All age groups	0.25	0.06	4.29	0.86–41.42	0.0516
*Throat tightness/pain*					
2–6 years	0.09	0.04	2.12	0.11–125.01	0.5928
7–10 years	1.26	0.00	—	—	—
All age groups	0.44	0.03	15.00	2.28–634.20	0.0003
*Hoarseness of voice*					
2–6 years	0.05	0.00	—	—	—
7–10 years	0.11	0.00	—	—	—
All age groups	0.06	0.00	—	—	—
*Shortness of breath*					
2–6 years	0.09	0.00	—	—	—
7–10 years	0.11	0.00	—	—	—
All age groups	0.09	0.00	—	—	—

^a^ Exposure‐adjusted incidence rate (EAIR) was calculated using treatment‐related Treatment Emergent Adverse Events (TEAEs), defined as adverse events judged by investigators to be possibly or probably related to study treatment, divided by the total treatment time in patient‐years.

^b^ Incidence rate ratio (IRR) was calculated with the EAIR of FOIT group divided by the EAIR of Placebo group; ratio cannot be calculated when either group has zero estimated event rate.

Dose adjustments during buildup affected 8 OIT and 5 placebo participants, with treatment‐related causes accounting for 75% of OIT episodes (failed updoses, pre‐updose symptoms, moderate‐to‐severe TEAEs) versus 40% in placebo. During maintenance, adjustments decreased (5 OIT, 2 placebo) and were primarily protocol‐mandated due to missed doses from travel or non‐allergic illnesses, suggesting improved tolerability at maintenance dosing (Table S13A).

At 52 weeks, OIT participants showed higher methylmercury levels [0.42 vs. 0.20 μg/L] reflecting increased fish consumption (Figure S8), but both groups remained within normal limits (< 1 μg/L) [9]. SCORAD eczema scores showed no significant between‐group differences (Figure S9). Urine levels of microplastics were comparable between treatment groups (Figure S10).

## 4 Discussion

To our knowledge, this study is the first double‐blind RCT to evaluate the efficacy and safety of codfish OIT intervention in young fish‐allergic children. As a prototype allergen, we selected codfish for this immunotherapy intervention given its status as both a commonly consumed fish species worldwide and one of the most prevalent fish allergens, with a well‐characterized allergen profile predominantly featuring parvalbumin (Gad c 1) as the major allergenic protein, making it an ideal representative model for investigating the broader therapeutic potential of fish OIT [10, 11].

Intention‐to‐treat analysis demonstrated that 52 weeks of fish OIT successfully induced desensitization in 43% of children compared with 11% receiving placebo (NNT = 4), with desensitized participants tolerating clinically meaningful doses of 13–17 g codfish protein (equivalent to 80–100 g fish). Desensitization was accompanied by significant reductions in fish‐specific IgE levels across multiple species (codfish, salmon, catfish), decreased skin prick test reactivity, reduced basophil activation and increased codfish‐specific IgG4 levels, confirming diminished IgE‐mediated allergic responses. Withdrawals and protocol deviations (e.g., post‐randomization dupilumab use) were addressed through sensitivity analyses. The robustness of our primary finding across these scenarios is reassuring, though it underscores a real‐world complexity in managing atopic comorbidities during extended trials. Our 52‐week protocol, though shorter than peanut OIT studies (≥ 24 months) [12, 13], achieved high retention (86% OIT, 94% placebo), demonstrating feasibility. The 1000 mg maintenance dose balanced efficacy and safety while minimizing potential environmental contaminant exposure in young children. Furthermore, the high challenge doses employed (13,330–17,663 mg cumulative protein) correspond to age‐appropriate, typical meal servings, establishing clinical relevance of our desensitization outcomes. The impact of extended treatment duration or increased maintenance dosing on desensitization rates remains to be determined.

Natural resolution of fish allergy occurs in only 15%–17% of children overall [14, 15], with tolerance developing in merely 3.4% of preschool children and 11.8% of school‐age children. Codfish allergy appears more persistent than other fish allergies, with a Greek study reporting that only 22% of affected children outgrew their allergies during follow‐up [4]. In this study, placebo participants demonstrated natural fluctuations in tolerated challenge doses between timepoints, particularly those with higher baseline reaction thresholds, with 11% (4/35) achieving desensitization at week 52 but only 9% (3/35) maintaining this after 8 weeks of avoidance. This indicates that naturally acquired tolerance can also be transient and unstable [16, 17, 18]. Our findings underscore the value of placebo controls in demonstrating therapeutic benefit beyond spontaneous improvement. Against this background of limited natural tolerance development, codfish OIT provides a pathway to greater clinical protection, with 69% of OIT participants achieving a 10‐fold increase in desensitization threshold to codfish compared to 26% in the placebo group. Thus, OIT offers a more effective approach than continued avoidance for achieving clinically meaningful protection against accidental exposures.

Similar to peanut OIT studies, our codfish OIT demonstrated characteristic immune remodeling with significant reductions in allergen‐specific IgE levels (codfish & rGad c 1, *p* < 0.001) and concurrent increases in blocking IgG4 antibodies (*p* < 0.001). These findings align closely with established mechanistic understanding from peanut and other food OIT trials, demonstrating consistent patterns of immune modulation across food allergens [19, 20, 21]. Notably, we observed immunological cross‐desensitization to salmon and catfish, extending beyond typical peanut OIT studies but consistent with conformational epitope recognition patterns [22, 23]. Our codfish OIT likely generates cross‐reactive antibodies targeting shared parvalbumin epitopes across fish species, analogous to cross‐reactive responses observed in tree nut OIT [24, 25]. Tree nut OIT studies demonstrate clinical cross‐desensitization through oral food challenges: walnut OIT achieved 100% pecan tolerance in co‐allergic patients, and cashew OIT similarly desensitized all pistachio co‐allergic participants [26, 27]. The mechanistic framework from tree nut studies—where memory B cell activation produces functionally suppressive IgG4 antibodies that compete with IgE for allergen binding—could potentially operate similarly in fish OIT, given parvalbumin's high sequence homology across species [28]. However, clinical cross‐desensitization remains to be demonstrated through formal oral food challenges with non‐codfish species.

Few OIT RCTs have assessed for SU in fish allergy, making our study among the first to evaluate this endpoint in a fish‐allergic population. Our trial employed an 8‐week secondary fish elimination period before reassessment, revealing that 23% of codfish OIT participants achieved SU compared to 9% in the placebo group. While this represents a modest improvement, it aligns with other food OIT trials reporting 30%–50% SU rates after 2–10 weeks of avoidance, indicating that SU remains challenging across food allergens [7, 12]. Exploratory subgroup analyses suggested age‐related differences in treatment response (Table S8B), though the unbalanced age distribution and limited power preclude definitive conclusions. Importantly, our immunological analysis revealed that participants achieving SU had markedly lower baseline allergen‐specific IgE to total IgE ratios compared to those achieving only desensitization (Figure 3B). After 8‐week avoidance, codfish‐specific IgG4 levels remained stable in the SU group while showing a decline in the DS group (*p* = 0.048), suggesting possible qualitative rather than quantitative antibody differences. We hypothesize that SU individuals represent a distinct immunological phenotype, potentially involving neutralizing, epitope‐specific IgG4 with IgE‐blocking activity mediated by regulatory cells [29, 30], while desensitization produces transient IgG4 lacking durable blocking function [29, 31]. Future studies should investigate the blocking activity and epitope specificity.

The safety profile of codfish OIT compares favorably with established benchmarks from other food OIT studies. Our epinephrine use rate of 3% in both groups falls at the lower end of typical OIT ranges (4%–9%) and below major peanut OIT trials (4%–13%) [7, 32]. Reactions concentrated during the build‐up phase (66% vs. 40% placebo) mirror other OIT trials where dose escalation represents the highest‐risk period [12]. The predominance of mild cutaneous and gastrointestinal symptoms, with low severe respiratory event rates (0.86–3.22 per person‐year), positions codfish OIT within acceptable risk–benefit profiles for food immunotherapy given the substantial desensitization benefits achieved. Furthermore, regular fish intake did not significantly increase the absorption of methylmercury and microplastics found in fish. However, the presence of microplastics in young children warrants further evaluation.

Several limitations should be acknowledged. First, our 52‐week treatment duration is shorter than most peanut OIT studies, which may have limited the SU rates, though the 43% full desensitization rate demonstrates meaningful immune modulation within 1 year. Our planned post‐treatment follow‐up will assess whether participants maintained their clinical response, experienced allergic reactions during real‐world fish consumption, and gained quality of life improvements [33]. Second, while immunological analyses demonstrated reduced cross‐reactivity to salmon and catfish allergens, clinical validation through OFCs with these fish species was not performed, leaving the clinical significance of this immunological cross‐reactivity unconfirmed. Third, treatment adherence was fair, with episodes of five or more consecutive missed doses occurring in 29%–31% of participants during buildup and 41%–43% during maintenance, primarily due to concurrent illnesses and travel. Notably, unlike peanut OIT where consecutive missed doses during buildup reduced desensitization rates [34], missed doses in our study did not significantly impact treatment outcomes. This adherence pattern, while comparable between groups, reflects inherent challenges of administering OIT in young children, including frequent respiratory infections requiring treatment suspension and the practical difficulties maintain regular fish intake during travel, potentially impacting assessment of true efficacy under optimal conditions. Fourth, the study population was skewed toward younger children (70% aged 2–6 years), which may limit generalizability to older age groups. Fifth, the potential impact of cooking methods on treatment outcomes warrants consideration. We standardized preparation by minimizing heating and freeze–thaw cycles to maintain parvalbumin stability, but we did not perform routine batch‐to‐batch quantification of Gad c 1 levels throughout the trial. We also did not assess whether variation among permitted cooking methods or family preferences affected clinical outcomes. Lastly, our study concentrated on a Chinese pediatric population with parvalbumin‐predominant sensitization, characteristic of cooked fish allergy. Whether findings can be extrapolated to other populations, particularly those with different fish consumption patterns (e.g., raw fish containing heat‐labile allergens like enolase and aldolase), remains uncertain. However, raw fish consumption is uncommon in pediatric populations.

In conclusion, this is the first double‐blind placebo‐controlled RCT of codfish OIT in young children with fish allergy, demonstrating desensitization in 43% of participants versus 11% receiving placebo (NNT = 4). The immunological findings indicate the successful modulation of fish‐specific immune responses, with potential cross‐species protection, representing a promising therapeutic approach given the persistence of fish allergy and the limited natural resolution rates. However, SU was achieved in only 23% of participants, indicating most children require ongoing treatment. Further studies with long‐term follow‐up and multi‐fish species validation are warranted before recommending codfish OIT for routine clinical practice.

## Author Contributions

Agnes Sze‐yin Leung has full access to all study data and takes responsibility for data integrity and analytical accuracy. Agnes Sze‐yin Leung contributed to study conceptualization, data collection, analysis and interpretation, and manuscript preparation and revision. Yanjun Gu and Ann Wing‐shan Au contributed to data collection and analysis. Rosetta Tsz‐ching Leung, Vanessa Hiu‐tung Tang, Kin Yi Fung, Janice Min Li contributed to data collection. Christine Yee‐yan Wai, Ting Fan Leung, and Gary Wing‐kin Wong contributed to expert opinion and scientific support. Members of the FOIT Working Group contributed to patient referral and clinical care. All authors participated in critical review, provided substantial intellectual contributions, and approved the final manuscript.

## Funding

This work was substantially supported by a grant from the Research Grants Council of the Hong Kong Special Administrative Region, China (Early Career Scheme reference no. 24109922). Additional support was provided by the State Key Laboratory of Marine Pollution (SKLMP) Seed Collaborative Research Fund 2023 and the Hong Kong Institute of Allergy Research Grant. The funder was not involved in the study design development, data collection procedures, analytical processes, interpretation of findings, or manuscript preparation.

## Conflicts of Interest

The authors declare no conflicts of interest.

## Supporting information


**Data S1:** all70268‐sup‐0001‐TableS1‐S16‐FigureS1‐S10.docx.

## Data Availability

The data that support the findings of this study are available on request from the corresponding author. The data are not publicly available due to privacy or ethical restrictions.
