# Supplementary material for: Codfish Oral Immunotherapy in Children Aged 2–10: Randomized Placebo‐Controlled Study
Source: Allergy. 2026 Feb 17;81(5):1799–812. doi: 10.1111/all.70268 (PMC13139779; doi:10.1111/all.70268)

**Supplementary Appendix**

**Study Title: Fish Oral Immunotherapy (FOIT) in Hong Kong Children**

[A randomised, controlled trial evaluating the effectiveness of Fish Oral Immunotherapy (FOIT) in inducing desensitisation or remission in children with fish allergy compared with placebo.]

**Corresponding author:** Dr. Agnes Sze-yin Leung, Department of Paediatrics, Faculty of Medicine, The Chinese University of Hong Kong.

Table of Contents

[Section I: Study Protocol 5](#_Toc216604531)

[- Aims and hypothesis 5](#_Toc216604532)

[- Methodology 5](#_Toc216604533)

[Study design 5](#_Toc216604534)

[Setting 5](#_Toc216604535)

[Participants and eligibility criteria 6](#_Toc216604536)

[Inclusion criteria 6](#_Toc216604537)

[Exclusion criteria 6](#_Toc216604538)

[Consent procedure 7](#_Toc216604539)

[Randomisation and concealment mechanism 7](#_Toc216604540)

[- Fish OIT 7](#_Toc216604541)

[Codfish selection 7](#_Toc216604542)

[Product preparation 7](#_Toc216604543)

[Rush induction visit (T0) — day 1 8](#_Toc216604544)

[Buildup Phase 9](#_Toc216604545)

[Maintenance Phase 9](#_Toc216604546)

[Elimination Phase 10](#_Toc216604547)

[Outcome Assessment 10](#_Toc216604548)

[Instructions to participants on fish avoidance / ingestion from end of treatment 11](#_Toc216604549)

[- Clinical endpoints 11](#_Toc216604550)

[Full desensitisation at T1 11](#_Toc216604551)

[Sustained unresponsiveness at T2 11](#_Toc216604552)

[- Study outcomes 12](#_Toc216604553)

[Primary Outcome 12](#_Toc216604554)

[Secondary Outcomes 12](#_Toc216604555)

[- Study visits 13](#_Toc216604556)

[- Study procedures 13](#_Toc216604557)

[Double Blind Placebo-Controlled Food Challenges (DBPCFC) 13](#_Toc216604558)

[- SPT and laboratory tests 15](#_Toc216604559)

[- Methodology for microplastic level quantification 17](#_Toc216604560)

[Urine Sample Collection and Microplastic Analysis 17](#_Toc216604561)

[- Participant compliance 18](#_Toc216604562)

[- Adverse events reporting 18](#_Toc216604563)

[- Dose modification 19](#_Toc216604564)

[- Missed doses 20](#_Toc216604565)

[- Statistical methods 21](#_Toc216604566)

[Sample size and power calculation 21](#_Toc216604567)

[Statistical analysis 21](#_Toc216604568)

[Primary & Secondary Outcomes 21](#_Toc216604569)

[- Patient and public involvement 22](#_Toc216604570)

[- Ethics and dissemination 22](#_Toc216604571)

[Section II: Fish selection as investigational products in Fish OIT 22](#_Toc216604572)

[- Selecting Fish Species 22](#_Toc216604573)

[- Sourcing and Sampling of Atlantic Codfish of Different Origins Available in Local Market 22](#_Toc216604574)

[- Mercury Analysis of Fish 25](#_Toc216604575)

[- Allergenicity by Cooking Method 26](#_Toc216604576)

[- Microbiological Quality after Frozen Storage 27](#_Toc216604577)

[- Summary of Findings 27](#_Toc216604578)

[Section III: Supplementary Trial Results 28](#_Toc216604579)

***List of Tables:***

- **Table S1. Dosing schedule for day 1 Rush Induction**
- **Table S2: Dosing Schedule for Buildup Phase (12 weeks)**
- **Table S3: Management for Adverse Reactions During Home OIT Dosing**
- **Table S4: DBPCFC - Food Challenge A and B for 2-6 years age group**
- **Table S5: DBPCFC - Food Challenge A and B for 7-10 years age group**
- **Table S6: Cessation criteria for DBPCFC and Rush induction, adopted from Chebar Lozinsky et al.**
- **Table S7. Details of Preparation Methods**
- **Table S8A: Treatment efficacy outcomes: desensitization and sustained unresponsiveness rates**
- **Table S8B: Exploratory subgroup analyses of desensitization (DS) and sustained unresponsiveness (SU) by age group (2-6 years vs. 7-10 years)**
- **Table S8C: Exploratory subgroup analyses of participant characteristics, desensitization (DS) and sustained unresponsiveness (SU) outcomes by presence of multiple food allergies.**
- **Table S9: Changes in immunological parameters from baseline to T1 between FOIT and placebo groups**
- **Table S10: Changes in immunological parameters from baseline to T1 between outcome groups**
- **Table S11A: Treatment emergent adverse events (TEAEs) by participant, categorized by causality**
- **Table S11B: Treatment emergent adverse events (TEAEs) by participant, categorized by severity ***
- **Table S12: Treatment emergent adverse events during the intervention period**
- **Table S13A: Overall Treatment Adherence Data (N=70 ITT Analysis)**
- **Table S13B: Comparison of Participants by Adherence: Those Missing ≥5 Doses vs <5 Doses**
- **Table S14 – Treatment adherence data during build-up phase**
- **Table S15 – Treatment adherence data during maintenance phaseTable S15 – Treatment adherence data during maintenance phase**
- **Table S16 – Withdrawal Rates and Reasons by Study Phase**

***List of Figures:***

- **Figure S1. Study flowchart**
- **Figure S2. Mercury in Codfish Samples of Different Origins**
- **Figure S3. 13.5% SDS-PAGE for Codfish Samples of Different Origins undergoing Different Cooking Methods**
- **Figure S4. Immunoblot with pooled sera from fish-allergic subjects (n=14)**
- **Figure S5 shows the probability of treatment success rates comparing codfish OIT to placebo groups and between desensitization and sustained unresponsiveness outcomes.**
- **Figure S6: Basophil activation responses.**
- **Figure S7: Codfish-specific antibody levels and IgG4/IgE ratios during the avoidance period.**
- **Figures S8: Methylmercury level in the red blood cells of codfish OIT vs placebo participants measured at week 52. Levels below 1 ng/ml are often considered normal for this age.**
- **Figure S9: Median (IQR) SCORAD scores of codfish OIT vs placebo participants during the treatment phase.**
- **Figure S10: Urinary microplastic levels in study participants.**

# Section I: Study Protocol

## Aims and hypothesis

This study tests the hypothesis that consumption of escalating subthreshold amounts of fish enhances immune tolerance in fish-allergic children and is safe and tolerable.

**Primary Aim:**

- To determine whether fish oral immunotherapy induces desensitization in children with fish allergy.

**Secondary Aims:**

- To evaluate the durability of treatment response following an avoidance period.
- To assess the safety profile of fish oral immunotherapy in children.
- To characterize immunological changes associated with successful treatment outcomes.

## Methodology

### Study design

A two-armed, randomised (1:1), stratified (by age: 2-6 and 7-10 years), double-blinded, placebo-controlled, parallel-group, superiority trial.

- Group 1 (FOIT) = Daily codfish OIT for 12 months
- Group 2 (Placebo) = Daily codfish-free placebo for 12 months.

The trial began on 22 Nov 2022.

### Setting

Conducted at Prince of Wales Hospital (PWH), Hong Kong, recruiting 70 participants from the PWH Paediatric Department and referrals.

Food challenges and treatment visits occur at PWH. Participants will self-administer interim immunotherapy doses at home. Participants receive an Anaphylaxis Action Plan, an epinephrine autoinjector, and training on handling allergic reactions. In the event of a reaction, they must adhere to their action plan and immediately contact the study team.

### Participants and eligibility criteria

Seventy children aged 2-10 years with confirmed codfish allergy — based on failed DBPCFC at study screening — will be randomised (n=35 per group). An additional 15 children will be enrolled as non-intervention reference controls.

These additional 15 non-interventional reference controls were enrolled to provide natural history data on fish allergy over time. These participants did not receive any study treatment (active or placebo) and were not included in the primary efficacy or safety analyses. Reference controls will be evaluated at T3 (12 months post-treatment completion, corresponding to approximately 24 months from baseline) to assess the natural course of fish allergy for comparison with treatment outcomes.

### Inclusion criteria

Subjects must meet all the following criteria:

- Aged 2 to 10 years. (Expanded from 3–10 years to include younger children for potential benefits of earlier intervention).
- Weight > 7kg (minimum weight safe for epinephrine autoinjector use).
- Confirmed codfish allergy at screening:
- Failed DBPCFC.
- Positive SPT (≥3mm wheal) or codfish-specific sIgE (≥0.35 kUA/L).

### Exclusion criteria

Subjects who meet any of the following criteria are not eligible:

- **Severe Anaphylaxis**: History or occurrence during DBPCFC of persistent hypotension, collapse, loss of consciousness, persistent hypoxia, or requiring >3 doses of intramuscular epinephrine or intravenous epinephrine infusion.
- **Biomarkers:** Serum codfish-specific IgE > 100 kUA/L
- **Respiratory Issues**: FEV1 <85%, FEV1/FVC <85%, or chronic persistent asthma.
- **High-Risk Conditions**: Cardiac disease, inflammatory intestinal conditions, immunocompromised states, post-surgery, critical illness, or prolonged hospitalization.
- **Medications**: Use of beta-blockers or ACE inhibitors.
- **Placebo Reaction**: Reaction to placebo during DBPCFC**.**
- **Recent Immunotherapy**: Food immunotherapy (<12 months) or current immunomodulatory therapy.
- **Major Illness:** Any condition that may pose risk or interfere with study participation, per Site Investigator judgment.
- **Eosinophilic esophagitis**: Suspected or biopsy-confirmed history.
- **Non-adherence**: Likely non-adherence to protocol, per Site Investigator judgement.

### Consent procedure

Parents/legal guardians will receive a CREC-approved Information Statement and Consent Form outlining study objectives, procedures, risks, and benefits before the screening visit. Before full enrolment, written consent from parent(s) must be obtained prior to collection of personal information, or procedures (e.g. screening DBPCFC).

### Randomisation and concealment mechanism

An independent statistician from the School of Public Health and Primary Care, CUHK generated the random allocation sequence. Eligible participants are enrolled by study personnel and assigned interventions using the randomisation list by an unblinded team. Unblinding of the securely-stored randomisation code is permitted only in emergencies, as judged by the Site Investigator, with documentation of the time, date, participant number, and reason.

## Fish OIT

### Codfish selection

Atlantic codfish (*Gadus morhua*) was selected for its dietary prevalence, abundance, and use as a model species in fish allergy research. Please refer to Section II for further details.

### Product preparation

Active FOIT doses contain varying doses of mashed, skinless, deboned codfish blended with potato, corn, carrot, onion, herbs, and spices. Placebo matches taste, texture, and appearance, but contains no fish. All products are prepared in a licensed kitchen compliant with Hong Kong Food Safety regulations, vacuum-sealed in individual packaging, stored below -18°C, transported with a maintained cold chain, and tested regularly for microbial safety (with aerobic plate count and *E. coli* testing on random samples). The IPs were dispensed to subjects in vacuum-sealed plastic packs, each containing 20 individual doses. Double-layer isothermal bags with ice packs were provided at IP pick-up by subjects or couriers to maintain the cold chain and maximize food safety during transport.

The investigational product (IP) was prepared from frozen codfish supplied by a single importer with arrangements to ensure proper cold chain maintenance throughout delivery. The fish underwent controlled thawing once for IP production, was then refrozen, and parents cooked the product from the frozen state following standardized instructions (baking, boiling, steaming, air-frying, or pan-frying at specified temperatures and durations; Table S7). This minimizes heating and freeze-thaw cycles to prevent potential structural changes and maintain parvalbumin stability.

### Rush induction visit (T0) — day 1

Participants receive escalating doses of FOIT (or placebo) every 20 minutes, up to a target of 120 mg of fish protein (cumulative 237mg), or until the highest tolerated dose (Table 1). Doses are administered as a potato mixture prepared by trained personnel independent of the study team.

If tolerated, they proceed to Buildup Phase the next day at 180mg (Dose 7). If a reaction occurs (as per predefined stopping criteria), they begin Buildup Phase at the last tolerated dose. Remaining Rush doses are incorporated into the Buildup Phase using a modified schedule. Subsequent dose increases will continue with the remaining Rush doses, followed by the standard Buildup Phase doses (Table 2).

##### Table S1. Dosing schedule for day 1 Rush Induction

| **Dose** | **Dosage**  Fish or placebo (mg) | **Dosage**  Fish protein or Placebo (mg) | **Cumulative** (mg) | **Percentage increase** (%) |
| --- | --- | --- | --- | --- |
| 1 | 25 | 3.7 | 3.7 | / |
| 2 | 60 | 9.0 | 12.7 | 200 |
| 3 | 100 | 15.0 | 27.7 | 200 |
| 4 | 200 | 30.0 | 57.7 | 200 |
| 5 | 400 | 60.0 | 117.7 | 200 |
| 6 | 800 | 120.0 | 237.7 | 200 |

### Buildup Phase

Daily fish OIT/ placebo doses increase every 2 weeks until reaching a ~1000mg maintenance dose. Dose increases may vary by +/- 7 days for scheduling flexibility.

Updoses are conducted under medical supervision. During COVID-19 restrictions, virtual medical supervision is used. If a reaction occurs, the participant will remain on the last tolerated dose.

Dietitians or parents prepare the doses. Parents will maintain daily diaries documenting dosing, adherence, reactions, and treatments administered.

##### Table S2: Dosing Schedule for Buildup Phase (12 weeks)

| **Dose** | **Dosage**  Fish or placebo (mg) | **Dosage**  Fish protein or placebo (mg) | **Percentage increase** (%) |
| --- | --- | --- | --- |
| 7 | 1200 | 180 | 150 |
| 8 | 1800 | 300 | 150 |
| 9 | 2700 | 450 | 150 |
| 10 | 3800 | 570 | 140 |
| 11 | 5400 | 900 | 142 |
| 12 | 6500 | 1000 | 120 |

### Maintenance Phase

Participants continue taking 1g of fish protein/ placebo daily at home for 12 months. Doses may be adjusted following predefined rules (Table 3).

##### Table S3: Management for Adverse Reactions During Home OIT Dosing

| **Non-anaphylactic Reactions:** |
| --- |
| - Maintain the current dose without changes for mild symptoms (e.g., urticaria, angioedema, vomiting, diarrhoea, abdominal pain). - Over-the-counter medications may be used for symptom relief. - Proceed with planned dose increases only if the participant has been symptom-free for at least 3 days prior. - Prophylactic antihistamines may be administered at the investigator’s discretion. - Delay dose increases by at least 7 days if allergic symptoms occur within 3 days of the scheduled updosing. |
| **Gastrointestinal Reactions:** |
| - Reduce the dose in cases of severe abdominal pain (scoring >6 on the Wong-Baker scale) lasting ≥20 minutes and occurring on 3 or more consecutive days. - Consider dose reduction if vomiting accompanied by abdominal pain recurs for 3 or more consecutive days. |
| **Anaphylactic Reactions:** |
| - Revert to the previous dose for at least 7 days following any anaphylaxis symptoms (e.g., stridor, wheezing, breathing difficulty, transient hypotension). - If the reaction occurs within 7 days of a dose increase: return to the previous dose until the next scheduled updose visit, then reattempt the dose that triggered the reaction. - If the reaction occurs 8 or more days after a dose increase: return to the previous dose until the next scheduled updose visit and maintain the reduced dose for an additional 2 weeks. |
| **Life-threatening Reactions:** |
| - Permanently discontinue OIT in cases of persistent hypotension, loss of consciousness, respiratory compromise requiring intubation, or the need for more than 3 doses of intramuscular epinephrine or an IV epinephrine infusion. - Participants will remain in the trial for follow-up through study completion. |

### Elimination Phase

Participants continue a fish-elimination diet for 8 weeks post-treatment.

### Outcome Assessment

Primary outcome analysis will occur once all participants have completed T1 and T2 DBPCFC, or withdrawn. Safety, tolerability, and secondary outcomes will be assessed at this time. A 6-month interim safety analysis is planned.

### Instructions to participants on fish avoidance / ingestion from end of treatment

The advice that is provided to participants regarding fish avoidance or ingestion upon completion of treatment will be as outlined below.

At T1, instructions will be provided based on the participant’s clinical status as outlined below:

- Subjects who passed T1 DBPCFC (Full desensitisation) will be arranged for T2 DBPCFC and will continue to avoid codfish (and other fish types) until the end of T2 Part B.
- Subjects who failed T1 DBPCFC at dose 6-8 will be advised to resume a regular intake of low dose of codfish (1 gram of codfish protein) at least 2 times per week, preferably daily, after 48 hours post-DBPCFC.
- Subjects who failed T1 DBPCFC at dose 1-5 will be advised to continue strict avoidance of codfish in their diet.

At T2, instructions will be provided based on the participant’s clinical status as outlined below:

- Subjects who passed T1 and T2 DBPCFC (Sustained unresponsiveness) will be advised to incorporate codfish into their diet ad libitum (preferably 1-2 serves of fish every 2-4 weeks).
- Subjects who passed T1 and failed T2 DBPCFC (Full desensitisation without Sustained unresponsiveness) will be commenced on a dose of codfish equivalent to ~7 grams codfish on the day following the completed T2 DBPCFC and instructed to continue this amount of codfish intake at least 2 times per week, preferably daily, until T3 evaluation (additional 12 months to maintain their desensitised stated).

## Clinical endpoints

### Full desensitisation at T1

Assessed via DBPCFC following completion of study treatment. Participants who pass the T1 DBPCFC are considered to achieve full desensitisation.

### Sustained unresponsiveness at T2

Assessed by a second DBPCFC eight weeks after treatment completion (T2). Only participants who pass the T1 DBPCFC will proceed to the T2 DBPCFC. SU is defined as successfully passing both T1 and T2 DBPCFCs.

## Study outcomes

### Primary Outcome

The success of the trial is solely determined by meeting the pre-defined statistical threshold for this endpoint, which is the proportion of fish-allergic subjects who achieve full desensitisation at T1. This is defined as tolerating a cumulative dose of:

- 13 grams fish protein (equivalent to 80 grams of fish) for participants aged 2-6 years.
- 17 grams fish protein (equivalent to 100 grams of fish) for participants aged 7-10 years.

### Secondary Outcomes

Proportion of participants achieving 8-week sustained unresponsiveness (passing both T1 and T2 DBPCFCs) in the active versus placebo groups.

- Cumulative dose tolerated during the T1 DBPCFC (defined as the dose at which a reaction occurs or the full challenge dose if no reaction occurs) in the active versus placebo groups.
- Change in fish SPT wheal size from baseline in active versus placebo groups at:
  - 6 months of treatment
  - End of treatment
  - 8 weeks post-treatment
- Change in serum/plasma levels of sIgE and sIgG4 associated with fish and its components at the same 3 timepoints above, in active versus placebo groups.
- Safety and tolerability, measured by the exposure-adjusted incidence rate and severity of treatment-emergent adverse events (TEAEs).

## Study visits as shown in Figure S1

##### Figure S1

**
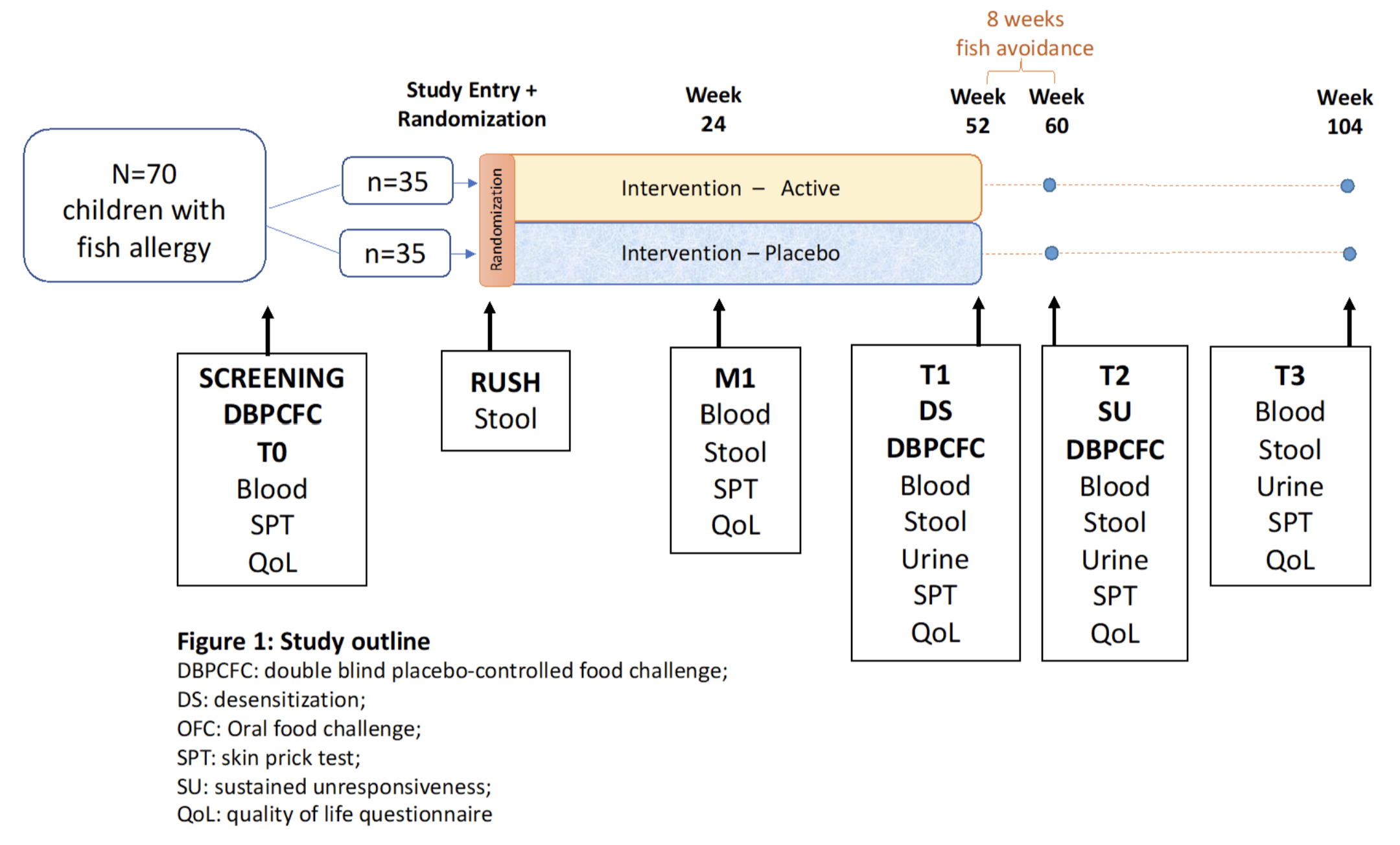
**

## Study procedures

### Double Blind Placebo-Controlled Food Challenges (DBPCFC)

DBPCFCs will be conducted at Screening, T1, and T2 in a hospital day ward over two separate days within a week. Each challenge consists of two parts (A and B), with one containing fish protein and the other placebo. Allocation of A and B will be randomised by the unblinded team. Study clinicians and nurses remain blinded. Nutritional services will prepare doses.

#### Total fish protein dose:

- - 13330mg (80g fish) for children aged 2-6 years
  - 17663 mg (106g fish) for children aged 7-10 years.
- Each part takes half a day, with ≥15-minute interval between doses. Participant are monitored for ≥2 hours post-challenge.
- An IV cannula will be inserted unless deemed unnecessary by the study physician.
- Spirometry will be performed for participants aged ≥8 years with asthma, or as clinically indicated.
- Challenge components will be unblinded only after both parts are completed, or if a reaction occurs to one part. (Tables 4, 5, 6).

#### DBPCFC Classification:

- **Passed**: No reaction to either part, allocation remains blind.
- **Failed:** Reaction to the fish component but not placebo, allocation unblinded.
- **Inconclusive:** Reaction to both or only to the placebo; allocation unblinded and challenge repeated. Inconclusive screening lead to exclusion from study.

##### Table S4: DBPCFC - Food Challenge A and B for 2-6 years age group

| **Dose** | **Weight of fish/ placebo (g)** | **Dose Fish protein/ placebo (mg)** | **Cumulative dose Fish protein/ placebo (mg)** |
| --- | --- | --- | --- |
| 1 | 0.06 | 10 | 10 |
| 2 | 0.2 | 30 | 40 |
| 3 | 0.6 | 90 | 130 |
| 4 | 1.8 | 300 | 430 |
| 5 | 5.4 | 900 | 1330 |
| 6 | 18 | 3000 | 4330 |
| 7 | 24 | 4000 | 8330 |
| 8 | 30 | 5000 | 13330 |

##### Table S5: DBPCFC - Food Challenge A and B for 7-10 years age group

| **Dose** | **Weight of fish/ placebo (g)** | **Dose Fish protein/ placebo (mg)** | **Cumulative dose Fish protein/ placebo (mg)** |
| --- | --- | --- | --- |
| 1 | 0.06 | 10 | 10 |
| 2 | 0.2 | 30 | 40 |
| 3 | 0.6 | 90 | 130 |
| 4 | 1.8 | 300 | 430 |
| 5 | 5.4 | 900 | 1330 |
| 6 | 18 | 3000 | 4330 |
| 7 | 36 | 6000 | 10330 |
| 8 | 44 | 7333 | 17663 |

##### Table S6: Cessation criteria for DBPCFC and Rush induction, adopted from Chebar Lozinsky et al.

| **Any of the following objective signs occurring within 2 hours of ingestion requires cessation:** |
| --- |
| - 3 or more concurrent non-contact urticaria persisting for at least 5 minutes. - Perioral, periorbital, or facial angioedema. - Vomiting (excluding gag reflex) and/or diarrhoea. - Persistent cough (i.e. not intermittent or transient throat clearing) - Wheeze (either audible (without stethoscope) or on auscultation with stethoscope), change in voice, stridor, difficulty breathing - Long bursts of sneezing/ persistent rhinorrhoea (persistent defined as on 3 or more doses or more than 40 minutes). - Collapse, hypotension - For Rush only, prolonged severe* abdominal pain for 40 minutes |
| **Any of the following requires delaying of the next scheduled dose (by 15 minutes for DBPCFC, or by 20 minutes for Rush)** |
| - Persistent throat tightness/ pain. - Severe* abdominal pain and/or notably distressed due to GI symptoms with decreased activity. - Mild subjective cardiovascular response (weak, dizzy) without evidence of hypotension or tachycardia. - < 3 non-contact urticaria or hard continuous scratching leading to excoriations. - Intermittent bursts of sneezing (<10), frequent sniffing. |
| * Severe abdominal pain defined as >6 on Wong-Baker FACES scale or a physician assessment of severity for younger children) |
| *Reference: Chebar Lozinsky A, Loke P, Orsini F, et al. Study protocol of a multicentre, randomised, controlled trial evaluating the effectiveness of probiotic and peanut oral immunotherapy (PPOIT) in inducing desensitisation or tolerance in children with peanut allergy compared with oral immunotherapy (OIT) alone and with placebo (the PPOIT-003 study). BMJ Open 2020;10(9):e035871. doi: 10.1136/bmjopen-2019-035871 [published Online First: 20200909]* |

## SPT and laboratory tests

Immunological evaluations were performed at screening, 6 months (M1), at the end of treatment (T1), and 8 weeks after treatment (T2). SPT was conducted using standardized allergen extracts including codfish (ALK US), fish mix consisting of codfish, halibut, and flounder (ALK US), salmon (ALK US), and catfish (Greer). Additional testing employed in-house produced extracts of grass carp (both raw and cooked preparations) and codfish (both raw and cooked preparations). Positive control utilized histamine (ALK Spain) and negative control employed normal saline. Blood samples collected at screening, T1 and T2 were used to measure the total IgE, codfish, salmon, catfish & rGad c 1-specific IgE and codfish-specific IgG4 levels by ImmunoCAP (Phadia AB, Uppsala, Sweden). Basophil activation was assessed by measuring CD63 expression upregulation using flow cytometry analysis. Plasma and PBMCs will be cryopreserved for future research. Stool and urine samples were collected at Rush induction (T0), M1, T1, T2.

**Basophil activation test (BAT):** Whole blood samples were collected from participants. BAT was conducted using the Flow CAST kit (BÜHLMANN Laboratories) with codfish (Gadus morhua) protein extract at 40 μg/ml at two timepoints: baseline before initial screening challenge (T0) and after 12 months of OIT (T1). Basophil activation was assessed through flow cytometric detection of CD63-expressing basophils on anti-FCεRI monoclonal antibody stimulation. Baseline and T1 BAT data with >5% in PB (high background activation) and <15% in PC1 (non-responder / invalid positive control) were excluded and not used in subsequent analyses.

**Methylmercury Analysis:** Methylmercury (MeHg) concentrations were analyzed using 50 μL aliquots from each blood sample. All samples underwent duplicate analysis as a minimum requirement, with additional replicate testing performed when the relative standard deviation (RSD) exceeded 20% to ensure analytical precision. Quality control was maintained using Seronorm Trace Elements Whole Blood L-3 Reference Use Only (RUO) as the standard reference material, with recovery values consistently falling within the 95% confidence interval for each analytical batch.

Blood samples underwent acid digestion with 4.6M nitric acid in a 60°C water bath for 16 hours. Following digestion, samples were pH-adjusted to 4.9 using acetate buffer and potassium hydroxide. Methylmercury was then ethylated in a closed purge vessel by adding sodium tetraethylborate, converting it to methylethyl mercury. The ethylated analogue was separated from solution by purging with argon gas onto a Tenax trap (porous polymer resin based on 2,6-diphenylene oxide). The trapped methylethyl mercury was thermally desorbed from the Tenax trap and transported via gas stream into a gas chromatography (GC) column for separation. Following isothermal GC separation, organo-mercury species entered a pyrolytic trap where they were converted to elemental mercury (Hg⁰) before quantification using cold vapor atomic fluorescence spectroscopy (CVAFS) detection.

## Methodology for microplastic level quantification

### Urine Sample Collection and Microplastic Analysis

Urine samples were collected using polypropylene (PP) Nalgene™ Wide-Mouth Lab Quality PPCO bottles (250 mL, Thermo Scientific™, catalog #2105-0008). Detailed instructions were provided for voiding and urine collection. After collection, containers were sealed tightly and returned to study personnel. All urine samples were processed within 2 hours of collection in a biosafety cabinet. Using a glass funnel to minimize plastic contamination, approximately 40 mL of urine was aliquoted into sterile 50 mL polypropylene conical centrifuge tubes (Nunc™, Thermo Scientific™, catalog #339653). Aliquoted samples were stored at -80°C within 15 minutes of processing, with processing time and storage location documented for each sample.

Each urine sample was collected in polypropylene containers and stored at −80°C prior to laboratory processing. After thawing, organic components were removed using the wet digestion protocol described by Li et al. (2002). A 10% hydrogen peroxide (H2O2; Sigma Aldrich, USA) solution was prefiltered through 0.22 µm pores and added to urine samples at a ratio of 1:2 (v/v, sample/ H2O2). The digestates were filtered using a vacuum pump connected to a filter funnel through a gold-coated polycarbonate membrane filters (Ø = 400 nm; Sterlitech, USA) with a filter area of 2 mm in diameter and rinsed three times with ultrapure water to ensure thorough removal of particles. Filter membranes were dried at room temperature and stored in glass Petri dishes until analysis.

To minimize contamination, all procedures were conducted within a fume hood. Glassware and tools were thoroughly rinsed with ultrapure water prior to use, and personnel wore cotton lab coats and nitrile gloves at all times. Ten procedural blank samples, consisting of ultrapure water in polypropylene containers, were processed alongside the urine samples to estimate background contamination. The mean microplastic (silicone) concentration in these blanks was 0.3 particles per 50 mL, which is equivalent to a background concentration of 0.006 particles/mL. Due to this low background level, no correction was applied to the final results.

Microplastics in the urine samples and procedural blank samples were analyzed using optical photothermal infrared (O-PTIR) spectroscopy with an mIRage IR microscope (Photothermal Spectroscopy Corp., Santa Barbara, CA, USA), equipped with a reflective Schwarzschild objective (×50, 0.78 NA). A tunable pulsed mid-IR quantum cascade laser (QCL; 1800–800 cm-1) induced photothermal effects through localized heating from radiation absorption, causing thermal expansion and refractive index changes in the sample. A continuous-wave single-frequency 532 nm visible laser, used as the probe beam, detected these photothermal effects. O-PTIR spectral measurements were conducted with the visible laser power set at 2.6% and IR power at 12%, using PTIR Studio software (v.4.4.8265, Photothermal Spectroscopy Corp.). For each sample, 10 mL of filtered urine was processed, and two randomly selected filter areas of 628 μm × 471 μm (approximately 18.83% of the total filter area) were scanned. The ChemicalID function in the mIRage software identified microplastic particles and determined polymer types with a hit quality index (HQI) of 80. Microplastic sizes were measured using the FeatureFinder function in the mIRage software.

Ref: Li, J., Qu, X., Su, L., Zhang, W., Yang, D., Kolandhasamy, P., Li, D. & Shi, H. (2016). Microplastics in mussels along the coastal waters of China. Environmental pollution, 214, 177-184.

## Participant compliance

Participants must bring treatments to every visit and maintain a daily diary documenting dose administration, reactions, and treatments used. Compliance will be assessed by diary reviews and treatment counts.

## Adverse events reporting

Participants report events in daily diaries, reviewed at each visit. At each visit, they are asked about well-being, hospitalisations, accidents, and medication and treatment changes. Physical examinations, laboratory results, or relevant medical records of adverse events are documented and assessed by the Principal Investigator or delegate for causality (unrelated, unlikely, possibly, or probably related), and severity (**allergic** or**non-allergic), using NIH NIAID Consortium system and ICH guidelines.**

Treatment-emergent adverse events (TEAEs) are presented both as the percentage of participants who experienced events and as exposure-adjusted incidence rates (EAIR), organized by specific event type and treatment assignment. EAIR was computed by dividing the total count of adverse events by the cumulative treatment duration expressed in patient-years. To enable direct comparison between treatment arms, we calculated incidence rate ratios (IRR) by dividing the FOIT group's EAIR by the Placebo group's EAIR; IRR could not be determined when either treatment group had no observed events. This methodology adjusts for varying exposure durations across treatment groups and provides a more precise evaluation of adverse event frequency throughout the study.

The definition of serious adverse events (SAEs) related to allergic reactions is based on the National Institutes of Health/National Institute of Allergy and Infectious Diseases (NIH/NIAID) consensus grading system. According to this grading system, episodes of anaphylaxis with persistent hypotension, collapse, loss of consciousness, or persistent hypoxia requiring intubation (Grade IV-V) are regarded as SAE.

## Dose modification

During the Buildup Phase, Updosing visits will be scheduled every 2 weeks +/- 7 days to accommodate parent/participant availability.

If *non-anaphylaxis* allergic symptoms develop following a dose of OIT taken at home (urticaria, angioedema, vomiting, diarrhoea, abdominal pain; without respiratory or cardiovascular involvement), the dose is continued without adjustment. Symptoms may be treated with over the counter medicines (e.g. antihistamine, pain reliever). The next scheduled dose increase will proceed uninterrupted provided the participant has been symptom free for at least 3 days prior to the scheduled Updose day. The participant may remain on antihistamine for prophylaxis against symptoms in the days leading up to the Updose day if the Site Investigator deems this to be warranted. If allergic symptoms (related to a dose of OIT) develop in the 3 days prior to a scheduled Updose day, the dose increase will not proceed and the current dose will be continued for a further 7 or more days or until the next scheduled Updose visit.

During home dosing, if severe abdominal pain (>6 on Wong Baker scale) lasting 20 mins or more, and recurring on 3 consecutive days or more, study doctors to consider reducing the dose to previous tolerated dose. If vomiting is associated with abdominal pain of any severity and recurs for 3 consecutive days or more, study doctors to consider reducing the dose to previous tolerated.

If *anaphylaxis symptoms* develop during home dosing (e.g. stridor, wheeze, difficulty breathing, transient hypotension), the immediate next dose will be reduced to the previous dose amount and continued for at least 7 days before proceeding with a dose increase. For example, if an anaphylaxis reaction to OIT occurs within the first 7 days of a dose increase, the daily dose will be reduced to the preceding dose amount for the remaining 7 or more days until the next scheduled Updose visit whereupon the dose will be increased back to the reaction-eliciting dose. However, if an anaphylaxis reaction to OIT occurs 8 or more days after a dose increase, the daily dose will be reduced to the preceding dose amount for the remaining 6 or fewer days until the next scheduled Updose visit whereupon the reduced daily dose will be repeated for a further 2 weeks (no dose increase).

If *life-threatening symptoms* of persistent hypotension, loss of consciousness, or respiratory compromise requiring intubation develop, or if a participant requires more than three doses of intramuscular epinephrine or an intravenous epinephrine infusion for management of an anaphylactic reaction, study treatment will be discontinued, however the participant will remain in the trial and will be followed-up through to the end of the study.

## Missed doses

If 1-4 consecutive days of OIT are missed, the participant may continue with the usual dose at home. If this occurs during the Buildup Phase, the next scheduled Updose may proceed as usual.

If 5-14 consecutive days of OIT are missed, OIT will be recommenced at the participant’s last tolerated dose and must be administered in hospital under medical supervision. If this occurs during the Buildup Phase, the daily dose must be taken for a minimum of 7 days before the next Updose can proceed.

If 15-28 consecutive days of OIT are missed, OIT will be recommenced at a reduced dose (the dose below the last tolerated dose) and must be administered in hospital under medical supervision. If this occurs during either the Buildup or Maintenance Phase, the reduced daily dose will be taken for a minimum of 2 weeks before the next Updose can proceed (Updosing schedule resumes or the dose is increased back to the maintenance dose, respectively).

If >28 consecutive days of dosing are missed, participants will recommence OIT at Day 1.

## Statistical methods

### Sample size and power calculation

The study sample size will be 70 participants, randomly allocated in a 1:1 ratio to active (n=35) and placebo (n=35). Based on literature, we estimate 15% of placebo to experience natural resolution of allergy, and 50% of active FOIT participants to achieve desensitization. With a significance level (α) = 0.05 and 80% power (two-tailed Pearson chi-squared test), 62 participants are required. To accommodate a conservative 30% dropout rate, the final sample size was increased to 70 (35 per group), providing an adjusted statistical power of 89%.

### Statistical analysis

Primary outcome analysis was performed upon completion of T2 DBPCFC assessments for all participants or study termination prior to T2 evaluation. Safety, tolerability, and secondary outcome analyses through the T2 timepoint were conducted concurrently. The safety population included all participants who received investigational product, regardless of completion status.

Data handling and analysis will be conducted at the Chinese University of Hong Kong in collaboration with an independent biostatistician, following an intention-to-treat (ITT) approach. Continuous variables will be summarized as **mean ± standard deviation** (or**median [interquartile range] for skewed data**), and categorical variables as **frequencies and percentages.** Statistical significance will be assessed using two-sided tests at the 5% level, with 95% CIs reported. Safety analyses will include all participants who received any investigational product.

### Primary & Secondary Outcomes

###

The study will be deemed successful if the primary efficacy comparison yields a p-value < 0.05, indicating a statistically significant higher rate of full desensitization in the OIT group compared to placebo. A 95% CI for the difference in desensitization rates will be calculated. The same statistical approach applies for the secondary outcome of sustained unresponsiveness. Logistic regression, adjusted for stratification variables such as age group, will be conducted, with odd ratios and corresponding 95% CIs reported.

## Patient and public involvement

Patients and the public were not engaged in the development of this study protocol.

## Ethics and dissemination

This study complies with the Declaration of Helsinki and ICH-GCP guidelines. Ethics approval was obtained from the Hong Kong CUHK-NTEC CREC (2011.666). Written informed consent will be obtained from participants' parents or guardians. Participation is voluntary, and withdrawal will not affect future care. Data and records will be anonymized using Study Identification Numbers (SID). Findings will be disseminated through peer-reviewed journals and international conferences.

# Section II: Fish selection as investigational products in Fish OIT

## Selecting Fish Species

Atlantic codfish (*Gadua morhua*) was selected as the fish species for OIT due to its popularity and abundance. Atlantic codfish has been traditionally used as a model in fish allergy research and its allergen as a representative marker for fish allergy diagnosis (de Martino et al., 1990). Our group has also reported that majority of the Chinese fish-allergic subjects were sensitized to codfish with sIgEs at the level of class 3 (3.5kUA/L) but lower than that to most freshwater fish species (Leung et al., 2024). Codfish was provided in varying sizes/doses, which was made from finely mashed skinless & deboned codfish in a mixture of potato, corn, carrot, onion, onion powder and thyme. Placebo was be made from potato, corn, carrot, onion, onion powder and thyme that had similar appearance, taste and smell to the active product. These fish and placebo products were also be used for screening, T1 and T2 DBPCFC.

## Sourcing and Sampling of Atlantic Codfish of Different Origins Available in Local Market

Frozen fillets from four countries of origin (Norway, Iceland, Canada, and Russia) were sourced from local suppliers and supermarkets. Fillets were randomly sampled for mercury analysis and prepared using six methods – raw, baking, air-frying, pan-frying, steaming, and boiling (Table S7) – for in vitro allergenicity evaluation by SDS-PAGE and immunoblotting. Samples were further stored in −20 °C freezer for 35 days and subsequently submitted for aerobic plate count (APC) and *Escherichia coli* (*E. coli*) testing to verify microbiological quality over the intended shelf-life of the investigational products.

Table S7. Details of Preparation Methods

| **Cooking Methods** | **Temperature/ Heat Level** | **Duration^*^** |
| --- | --- | --- |
| 1. Baking | 150°C | 10min 30sec |
| 2. Boiling | In 100°C boiling water | 3min |
| 3. Steaming | Over 100°C boiling water | 3min |
| 4. Air-frying | 150°C | 3min 30sec |
| 5. Pan-frying | Medium heat | 2min 40sec |
| * The cooking durations were determined by the time required for the central temperature of a 30g codfish block to reach 65°C, measured with a calibrated probe thermometer – slightly above the USDA’s recommended safe minimum internal temperature for fish (62.8°C; <https://www.fsis.usda.gov/food-safety/safe-food-handling-and-preparation/food-safety-basics/safe-temperature-chart>). | | |

| 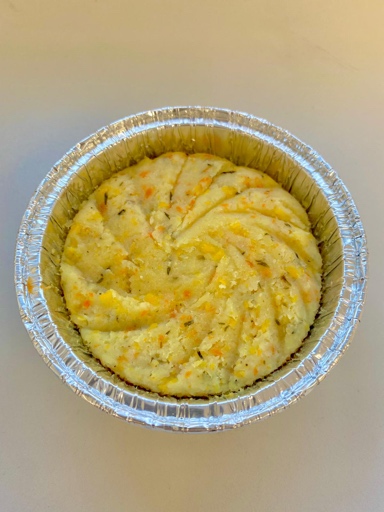 | 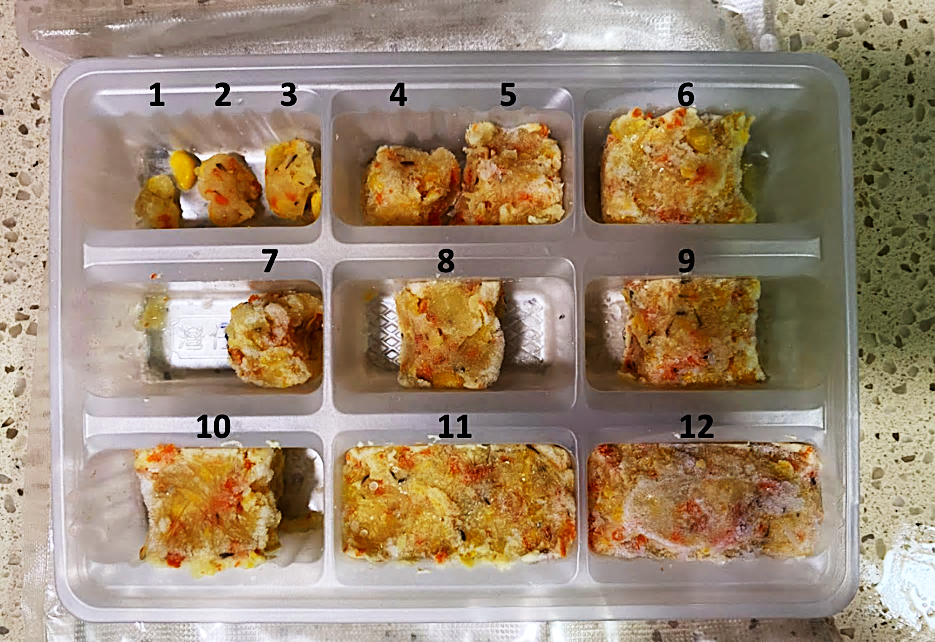 |
| --- | --- |
| Codfish (placebo) served at DBPCFCs (above) | Daily home dosing of codfish OIT product or placebo (doses 7-12 shown above) |

Dose 12 represents the maintenance dose containing 1000mg fish protein (equivalent to 6.5g codfish). When blended with the vehicle (potato, corn, and seasonings), the total weight is approximately 30g (shown above), which can be consumed in 2-3 mouthfuls by children and is typically incorporated into main meals. Dimensions of the fish used for DBPCFC are shown below.

Amount of fish in double-blind placebo-controlled food challenges

| **D1: 0.06 g** | **D2: 0.2 g** | | | **D3: 0.6 g** | | **D4: 1.8 g** | | | **D5: 5.4 g** |
| --- | --- | --- | --- | --- | --- | --- | --- | --- | --- |
| 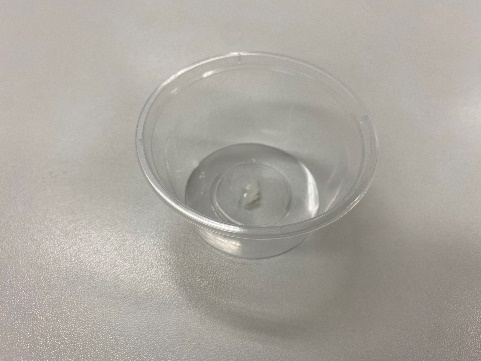 | 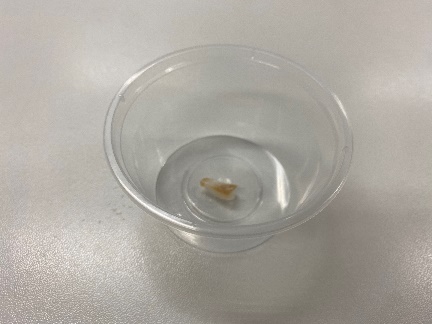 | | | 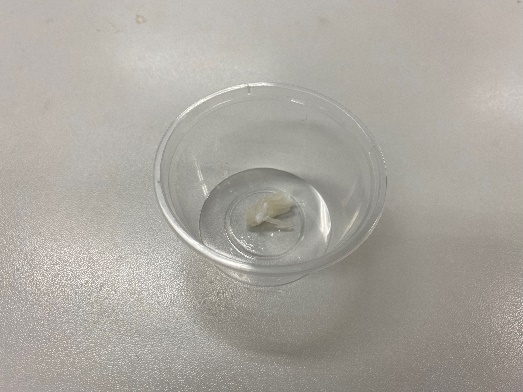 | | 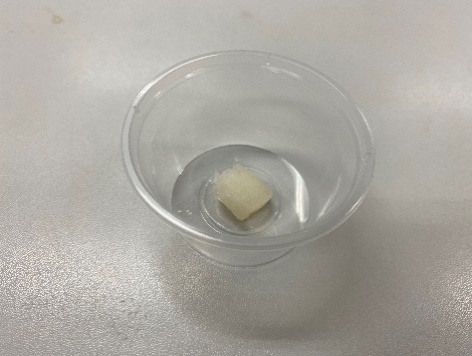 | | | 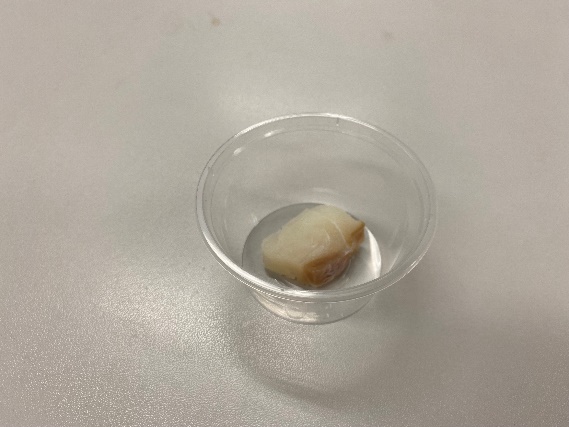 |
| **D6: 18 g** | | **[2-6yo] D7: 24 g** | | | **[2-6yo] D8: 30 g** | | | **[2-6yo] Total: 80.06 g** | |
| 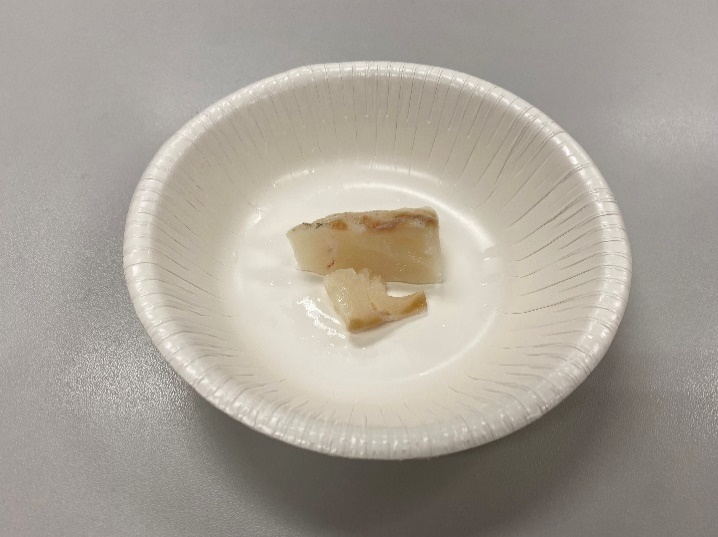 | | 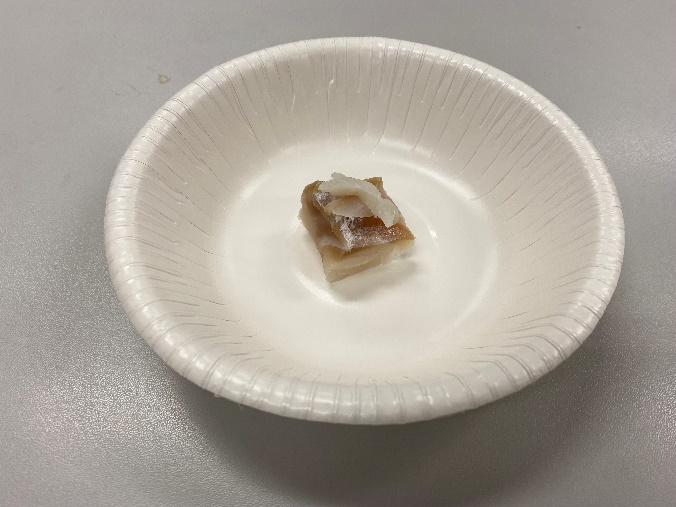 | | | 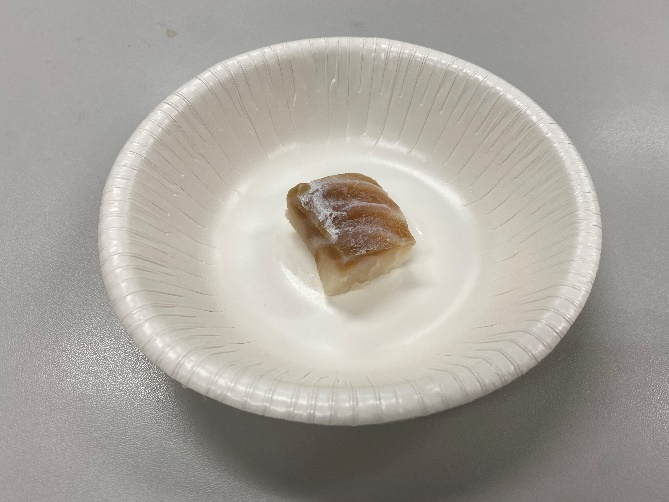 | | | 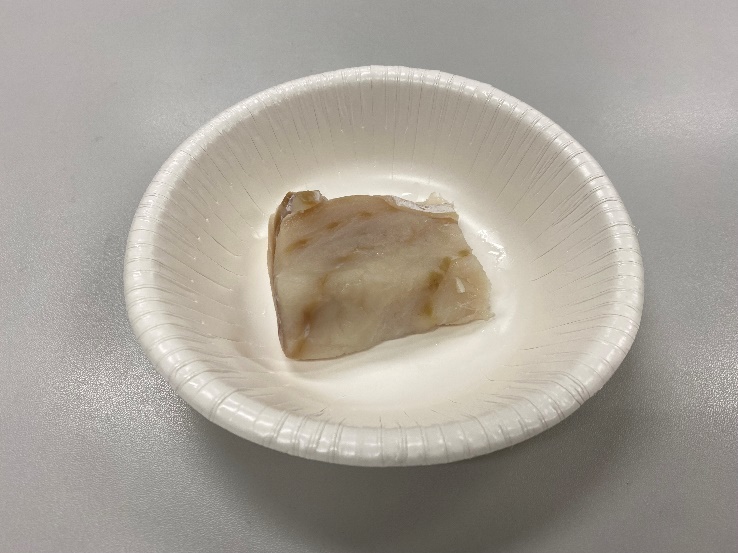 | |
| **[7-10yo] D7: 36 g** | | | **[7-10yo] D8: 44 g** | | | | **[7-10yo] Total: 106.06 g** | | |
| 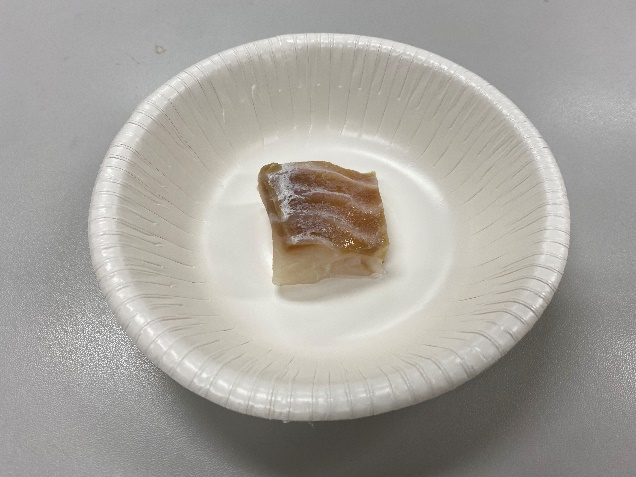 | | | 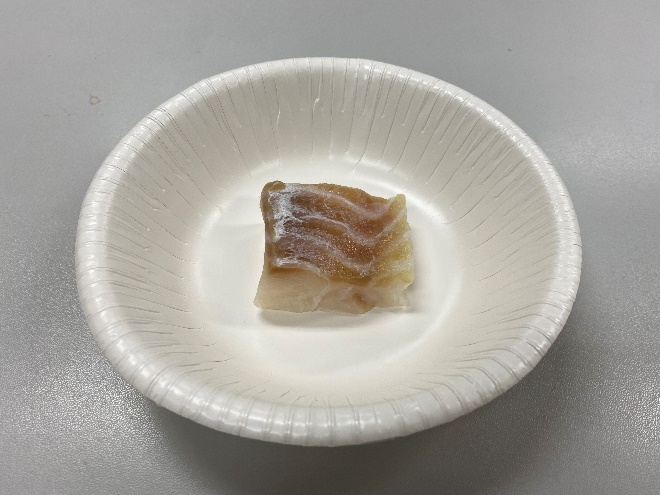 | | | | 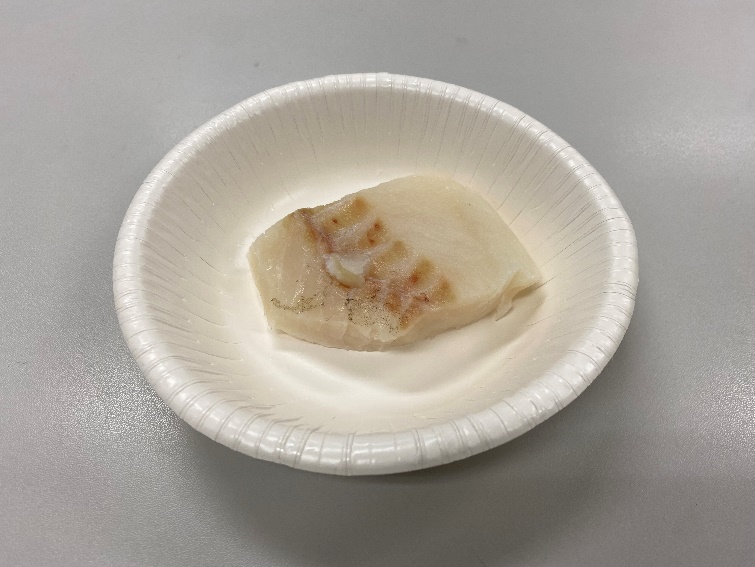 | | |

## Mercury Analysis of Fish

Codfish originating from Canada was tested with the highest mercury content (mean 396 +/- 7.6µg/kg), which was double of that from Norway (mean 183 +/- 2.7µg/kg), and almost triple of those from Iceland (mean 143 +/- 2.5µg/kg) and Russia (mean 134 +/- 2.2µg/kg)(Figure S2).

##### Figure S2. Mercury in Codfish Samples of Different Origins


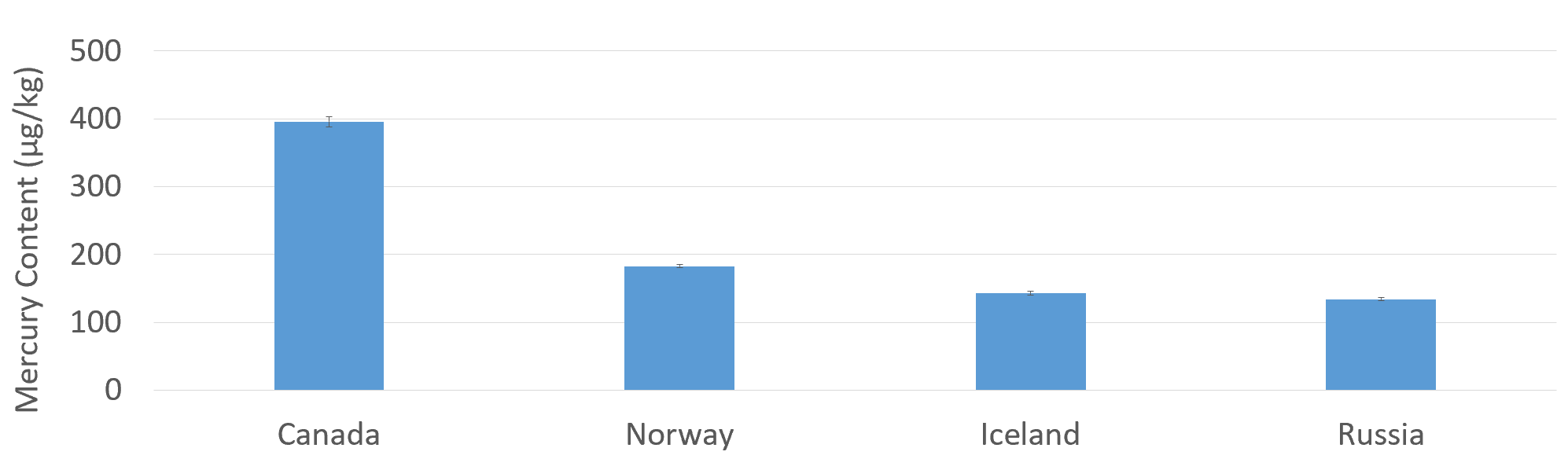


The serving portion is in line with recommendation from the European Food Safety Authority (EFSA) guideline (max 1.3 μg/kg bw/week) on the safe consumption of fish. Below shows the recommended maximum codfish consumption per week (g/week=1.3*bw/0.584):

| Age (year) | 3 | 4 | 5 | 6 | 7 | 8 | 9 | 10 | 11 |
| --- | --- | --- | --- | --- | --- | --- | --- | --- | --- |
| Body weight (kg) | 13.3 | 15.0 | 17.0 | 19.0 | 21.3 | 24.0 | 27.0 | 30.3 | 34.0 |
| Max intake (g/week) at 0.584 μg/g based on previous local data (P.H.Y.Chan et al., 2018) | 29.5 | 33.4 | 37.8 | 42.3 | 47.3 | 53.4 | 60.1 | 67.3 | 75.7 |
| **Max intake (g/week) at 0.183 μg/g based on our own data as shown above** | **94.4** | **106.6** | **120.8** | **135.0** | **151.2** | **170.5** | **191.8** | **215.1** | **241.5** |

Daily consumption of 6.5g codfish (equivalent to 45.5g per week) falls well within safe mercury exposure limits for young children based on our measured mercury content of 0.183 μg/kg in the study fish. Our maintenance dose of 45.5g per week represents only 48% of the safe limit for the youngest participants (3 years old) and progressively smaller percentages for older children (e.g., 21% for 11-year-olds).

## Allergenicity by Cooking Method

SDS-PAGE showed higher content of parvalbumin (PV) in raw extracts of Norwegian and Canadian codfish samples. Air-frying and pan-frying retained more PV and other proteins, whereas baking and boiling led to greater loss of PV (Figure S3). Immunoblot showed similar results, with relative PV allergenicity of 1.0 (raw), 0.34 (bake), 0.48 (airfry), 0.38 (panfry), 0.42 (steam) and 0.40 (boil) (Figure S4).

##### Figure S3. 13.5% SDS-PAGE for Codfish Samples of Different Origins undergoing Different Cooking Methods


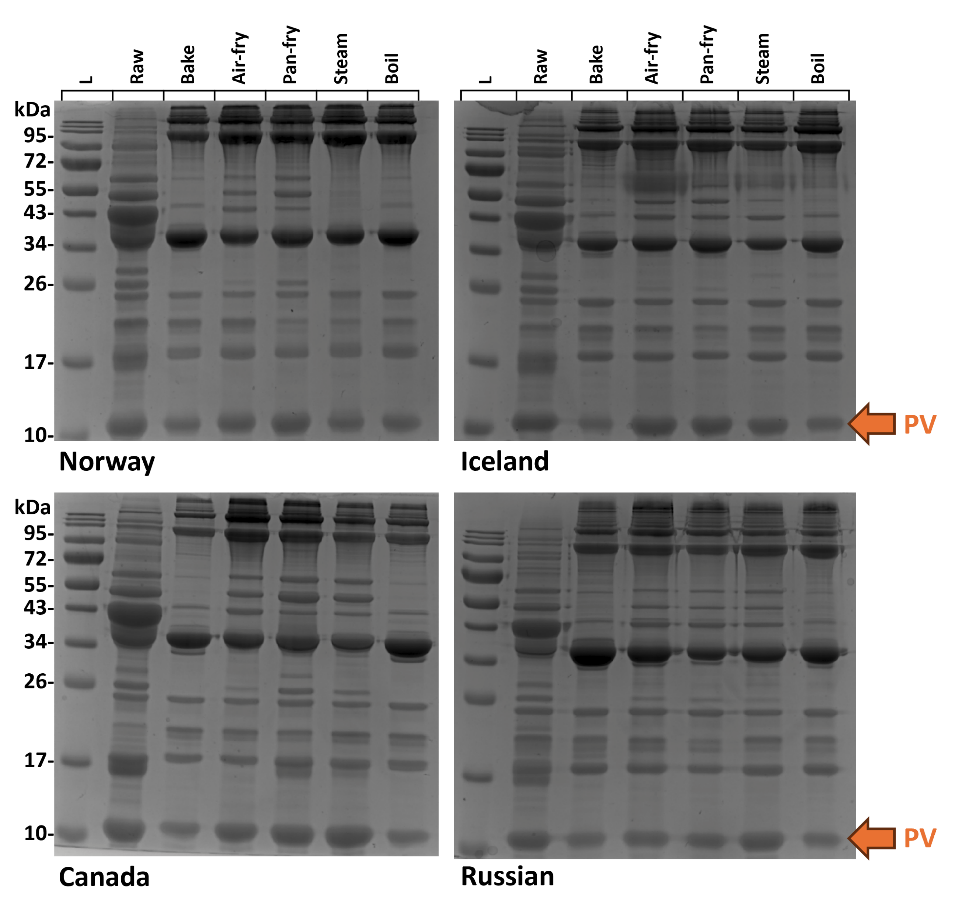


#####

##### Figure S4. Immunoblot with pooled sera from fish-allergic subjects (n=14)


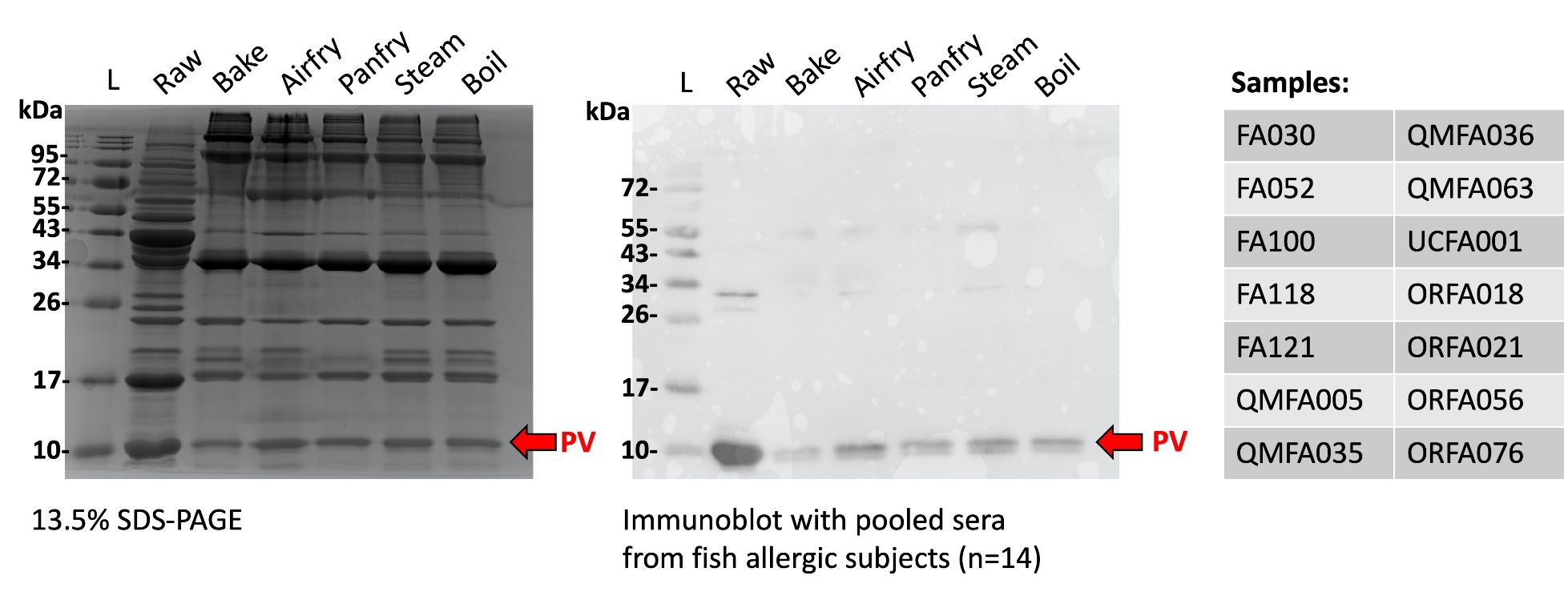


## Microbiological Quality after Frozen Storage

Samples stored at −20 °C for 35 days met microbiological criteria:

- Active: APC 470 cfu/g; *E. coli* not detected
- Placebo: APC 160 cfu/g; *E. coli* not detected
- Reference limits: APC < 1,000 cfu/g; *E. coli* < 20 cfu/g

  (Reference from *Centre for Food Safety (2014). Microbiological Guidelines for Food. Centre for Food Safety, Food and Environmental Hygiene Department, HKSARG.*)

## Summary of Findings

Norwegian codfish appeared the most suitable for use as the investigational product for codfish OIT, combining relatively low mercury levels with higher PV content. Although air-frying and pan-frying preserved more PV, air-frying may be less practical for many households. Steaming was recommended as an alternative cooking method for preparing the home dosing.

# Section III: Supplementary Trial Results

Besides ITT and PP analyses, multiple sensitivity analyses were conducted, including:

The table below summarizes how different participant categories were handled across analytical approaches:

| **Analysis** | **Dupilumab Case** | **Inconclusive*** | **Active Withdrawal** | **Placebo Withdrawal** |
| --- | --- | --- | --- | --- |
| **ITT** | Success | Failed | Failed | Failed |
| **Per-Protocol** | Excluded | Excluded | Excluded | Excluded |
| **Sensitivity 1** | Failed | Failed | Failed | Success |
| **Sensitivity 2** | Failed | Failed | Failed | Failed |
| **Sensitivity 3** | Success | Failed | Failed | Success |

* The inconclusive case reacted to both placebo and active fish during the end-of-treatment DBPCFCs; the challenges were repeated and the participant reacted on both days again. Per protocol, these were excluded as they are considered to have no primary outcome data available.

Rationale for each approach:

- ITT: Preserves randomization and includes all participants as assigned, counting the dupilumab participant's observed outcome (achieved sustained unresponsiveness)
- Per-Protocol: Includes only participants who completed the study according to protocol requirements without major violations
- Sensitivity 1 **(Extreme Worst-Case Scenario for the Treatment Group)**: Worst-case scenario for FOIT—assumes dupilumab case failed and optimistically counts placebo withdrawals as successes
- Sensitivity 2 **(A More Neutral (But Still Conservative) Scenario)**: Conservative scenario— assumes dupilumab case failed and all withdrawals failed in both groups
- Sensitivity 3 **(A Mixed but Realistic Scenario)**: Credits the observed dupilumab outcome while optimistically counting placebo withdrawals as successes

#####

##### Table S8A: Treatment efficacy outcomes: desensitization and sustained unresponsiveness rates

|  | **Probability of Desensitization**  **(95% CI)**  **P-value** | **Probability of Sustained Unresponsiveness (95% CI)**  **P-value** |
| --- | --- | --- |
| ITT | FOIT: 0.43 (0.26, 0.60)  Placebo: 0.11 (0.00, 0.23)  P = 0.003  Difference: 0.32 (0.09, 0.51)  NNT: 4 (2, 12) | FOIT: 0.23 (0.10, 0.41)  Placebo: 0.09 (-0.01, 0.19)  P = 0.332  Difference: 0.14 (-0.05, 0.33)  NNT: 7 |
| PP | FOIT: 0.52 (0.32, 0.71)  Placebo: 0.12 (0.00, 0.24)  P < 0.001  Difference: 0.40 (0.14, 0.60)  NNT: 3 (2, 7) | FOIT: 0.28 (0.10, 0.45)  Placebo: 0.09 (-0.01, 0.19)  P = 0.094  Difference: 0.19 (-0.03, 0.40)  NNT: 6 |
| Sensitivity 1 | FOIT: 0.40 (0.23, 0.57)  Placebo: 0.17 (0.04, 0.30)  P = 0.063  Difference: 0.23 (0.00, 0.43)  NNT: 5 | FOIT: 0.20 (0.06, 0.34)  Placebo: 0.14 (0.02, 0.26)  P = 0.752  Difference: 0.06 (-0.14, 0.25)  NNT: 18 |
| Sensitivity 2 | FOIT: 0.40 (0.23, 0.57)  Placebo: 0.11 (0.03, 0.23)  P = 0.013  Difference: 0.29 (0.06, 0.48)  NNT: 4 (2, 17) | FOIT: 0.20 (0.06, 0.34)  Placebo: 0.09 (-0.01, 0.18)  P = 0.306  Difference: 0.11 (-0.08, 0.30)  NNT: 9 |
| Sensitivity 3 | FOIT: 0.43 (0.26, 0.60)  Placebo: 0.17 (0.04, 0.30)  P = 0.036  Difference: 0.26 (0.02, 0.46)  NNT: 4 (2, 46) | FOIT: 0.23 (0.08, 0.37)  Placebo: 0.14 (0.02, 0.26)  P = 0.540  Difference: 0.09 (-0.12, 0.28)  NNT: 12 |

##### Table S8B: Exploratory subgroup analyses of desensitization (DS) and sustained unresponsiveness (SU) by age group (2-6 years vs. 7-10 years)

| Outcome | Age group  (years) | FOIT  n/N (%) | Placebo  n/N (%) | Risk difference  (95% CI) | Fisher’s  p value |
| --- | --- | --- | --- | --- | --- |
| **DS** | 2–6 | 9/25 (36.0) | 1/24 (4.2) | 31.8% (0.06, 0.54) | 0.011 |
|  | 7–10 | 6/10 (60.0) | 3/11 (27.3) | 32.7% (-0.14, 0.66) | 0.198 |
| **SU** | 2–6 | 5/25 (20.0) | 0/24 (0.0) | 20.0% (-0.01, 0.41) | 0.050 |
|  | 7–10 | 3/10 (30.0) | 3/11 (27.3) | 2.7% (-0.37, 0.43) | 1.000 |

##### Table S8C: Exploratory subgroup analyses of participant characteristics, desensitization (DS) and sustained unresponsiveness (SU) outcomes by presence of multiple food allergies.

|  | **Multiple FA**  **(n = 42)** | **Non-multiple FA**  **(n = 28)** | **P value** |
| --- | --- | --- | --- |
| DS, n (%) | 10 (24) | 9 (32) | 0.584 |
| SU, n (%) | 5 (12) | 6 (21) | 0.328 |
| Months of age, median (IQR) | 63 (39, 88) | 68 (39, 95) | 0.458 |
| **Baseline, median (IQR)** | | | |
| Total IgE | 788 (464, 1805) | 853 (270, 1196) | 0.365 |
| sIgE (codfish) | 4.4 (1.5, 10.6) | 1.9 (0.8, 7.2) | 0.057 |
| sIgE (rGad c1) | 18.1 (4.5, 29.4) | 5.9 (3.8, 25.5) | 0.211 |
| sIgG4 (codfish) | 0.6 (0.1, 1.9) | 0.4 (0.2, 1.0) | 0.701 |
| SPT (codfish) | 5.0 (3.5, 6.5) | 4.0 (3.0, 4.5) | **0.005** |
| BAT (codfish) | 72.0 (32.2, 83.8) | 42.0 (7.3, 75.1) | **0.046** |
| **T1, median (IQR)** | | | |
| Total IgE | 1088 (415, 2015) | 799 (296, 1891) | 0.740 |
| sIgE (codfish) | 2.1 (1.3, 6.0) | 1.2 (0.9, 3.6) | **0.034** |
| sIgE (rGad c1) | 9.1 (3.5, 26.4) | 4.2 (2.8, 10.6) | 0.068 |
| sIgG4 (codfish) | 1.7 (0.4, 4.6) | 0.6 (0.2, 2.0) | 0.054 |
| SPT (codfish) | 3.5 (2.5, 5.3) | 3.5 (2.4, 5.6) | 0.511 |
| BAT (codfish) | 56.3 (30.2, 67.9) | 67.6 (36.9, 92.4) | 0.419 |

##### Figure S5 shows the probability of treatment success rates comparing codfish OIT to placebo groups and between desensitization and sustained unresponsiveness outcomes.


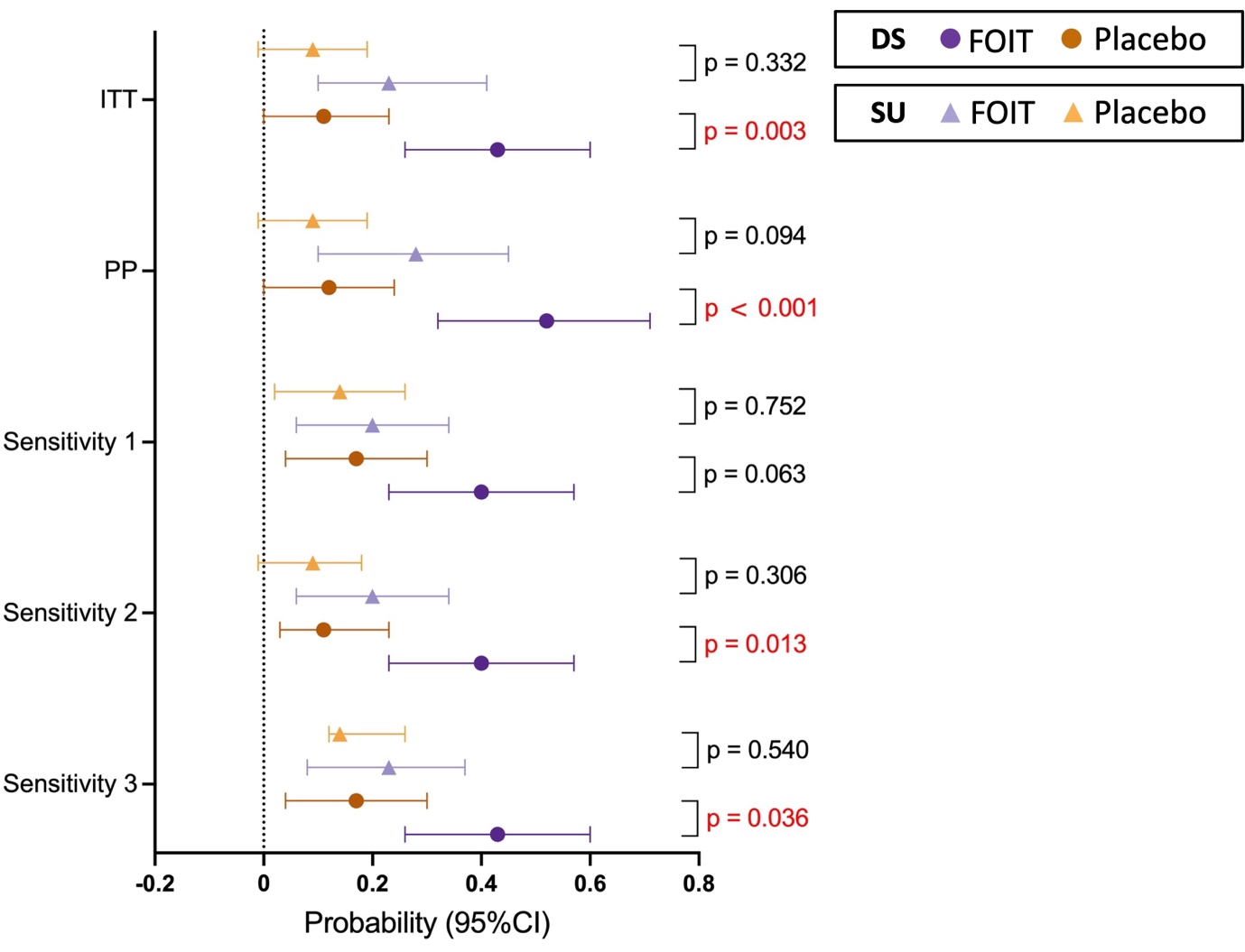


##### Table S9: Changes in immunological parameters from baseline to T1 between FOIT and placebo groups

| Median (IQR) | FOIT | | | Placebo | | |
| --- | --- | --- | --- | --- | --- | --- |
|  | **Baseline** | **T1** | **p value** | **Baseline** | **T1** | **p value** |
| Codfish sIgE (kUA/L) | 4.4 (1.5, 10.2) | 1.2 (0.4, 3.1) | **< 0.001** | 3.0 (0.9, 6.3) | 2.5 (1.2, 6.7) | 0.977 |
| log10(Codfish sIgE/Total IgE) | -2.3 (-2.6, -1.9) | -2.7 (-3.0, -2.2) | **< 0.001** | -2.6 (-2.9, -2.2) | -2.5 (-2.9, -2.3) | 0.271 |
| rGad c 1 sIgE (kUA/L) | 11.4 (5.0, 26.3) | 4.5 (2.4, 10.5) | **< 0.001** | 13.1 (4.5, 34.6) | 9.2 (3.2, 35.5) | 0.207 |
| log10(rGad c 1 sIgE/Total IgE) | -1.8 (-2.2, -1.4) | -2.2 (-2.7, -1.7) | **< 0.001** | -1.8 (-2.5, -1.5) | -1.8 (-2.4, -1.5) | 0.064 |
| Codfish sIgG4 (mgA/L) | 0.6 (0.1, 1.8) | 2.4 (0.9, 5.2) | **< 0.001** | 0.4 (0.1, 1.4) | 0.5 (0.1, 1.4) | 0.471 |
| log10(Codfish sIgG4/Codfish sIgE) | -0.9 (-1.6, -0.3) | 0.1 (-0.1, 0.5) | **< 0.001** | -0.7 (-1.4, -0.2) | -1.0 (-1.3, -0.1) | 0.900 |
| Codfish SPT (mm) | 5.0 (3.5, 6.5) | 2.5 (2.0, 3.6) | **< 0.001** | 4.5 (3.5, 5.5) | 4.5 (3.3, 6.5) | 0.805 |
| Codfish_BAT (CD63%) | 66.4 (36.3, 85.6) | 7.7 (5.8, 75.3) | **0.016** | 68.1 (7.3, 79.5) | 48.5 (1.2, 78.0) | 0.517 |
| Salmon sIgE (kUA/L) | 2.5 (1.3, 7.3) | 1.5 (0.6, 4.3) | **< 0.001** | 3.7 (0.8, 11.2) | 3.1 (0.9, 15.4) | 0.599 |
| Catfish sIgE (kUA/L) | 6.2 (3.3, 19.0) | 3.2 (2.1,8.7) | **< 0.001** | 9.6 (3.5, 17.7) | 7.6 (3.1, 23.6) | 0.143 |

##### Table S10: Changes in immunological parameters from baseline to T1 between outcome groups

| Median (IQR) | SU (FOIT+Placebo) | | | DS (FOIT+Placebo) | | | Partial desensitization (FOIT+Placebo) | | | Persistent allergy (FOIT+Placebo) | | |
| --- | --- | --- | --- | --- | --- | --- | --- | --- | --- | --- | --- | --- |
|  | **Baseline** | **T1** | **p value** | **Baseline** | **T1** | **p value** | **Baseline** | **T1** | **p value** | **Baseline** | **T1** | **p value** |
| Codfish sIgE (kUA/L) | 1.9 (0.3, 4.0) | 0.6 (0.2, 1.6) | **0.010** | 6.1 (2.4, 10.2) | 3.3 (1.4, 5.3) | **0.023** | 3.6 (1.3, 8.8) | 2.7 (1.2, 5.7) | 0.125 | 2.5 (0.9, 12.0) | 1.8 (1.1, 7.4) | 0.212 |
| log10(Codfish sIgE/Total IgE) | -2.8 (-3.3, -2.5) | -3.0 (-2.4, -2.8) | **0.002** | -2.3 (-2.5, -1.9) | -2.5 (-2.9, -2.1) | **0.016** | -2.3 (-2.7, -1.9) | -2.4 (-2.7, -2.2) | **0.022** | -2.5 (-3.0, -2.1) | -2.5 (-2.9, -2.2) | 0.132 |
| rGad c 1 sIgE (kUA/L) | 5.6 (1.1, 10.4) | 3.6 (1.0, 4.5) | **0.002** | 22.7 (6.6, 31.8) | 12.7 (4.2, 19.6) | **0.016** | 13.0 (4.2, 27.8) | 8.4 (3.0, 31.0) | 0.074 | 6.5 (4.1, 37.0) | 7.3 (3.3, 29.7) | 0.067 |
| log10(rGad c 1 sIgE/Total IgE) | -2.5 (-2.6, -2.0) | -2.7 (-3.1, -2.2) | **< 0.001** | -1.9 (-2.0, -1.4) | -1.9 (-2.4, -1,5) | **0.016** | -1.7 (-2.0, -1.4) | -1.8 (-2.3, -1.5) | **0.033** | -1.8 (-2.5, -1,4) | -1.8 (-2.4, -1.5) | **0.039** |
| Codfish SPT (mm) | 4.5 (3.5, 6.5) | 2.0 (2.0, 4.0) | **0.020** | 5.5 (4.3, 7.1) | 2.8 (1.8, 3.4) | **0.031** | 4.5 (3.3, 5.8) | 3.5 (2.8, 6.3) | 0.410 | 4.0 (3.5, 5.0) | 4.8 (3.0, 6.1) | 1.000 |
| Codfish sIgG4 (mgA/L) | 0.4 (0.1, 3.2) | 1.7 (0.5, 4.5) | 0.175 | 0.6 (0.2, 2.0) | 4.2 (1.7, 6.4) | 0.055 | 0.8 (0.2, 1.5) | 1.1 (0.2, 3.4) | 0.082 | 0.3 (0.0, 0.7) | 0.3 (0.1, 1.0) | 0.121 |
| log10(Codfish sIgG4/Codfish sIgE) | -0.4 (-1.6, 0.2) | 0.4 (-0.1,0.8) | 0.083 | -1.0 (-1.8, -0.5) | 0.1 (-0.2,0.2) | 0.195 | -0.6 (-1.5, 0.0) | -0.2 (-1.2, 0.2) | **0.014** | -1.1 (-1.6, -0.7) | -1.1 (-1.3, -0.1) | **0.026** |

##### Figure S6: Basophil activation responses.

Paired baseline and post-treatment samples were available for a subset of participants (FOIT: n = 25 baseline, n = 14 at T1; Placebo: n = 21 baseline, n = 28 at T1) due to limited sample collection. In participants with paired measurements, codfish basophil reactivity significantly decreased from baseline to the end of treatment (median 66.4% vs. 7.7%, p = 0.013), but the change was non-significant for the placebo group.


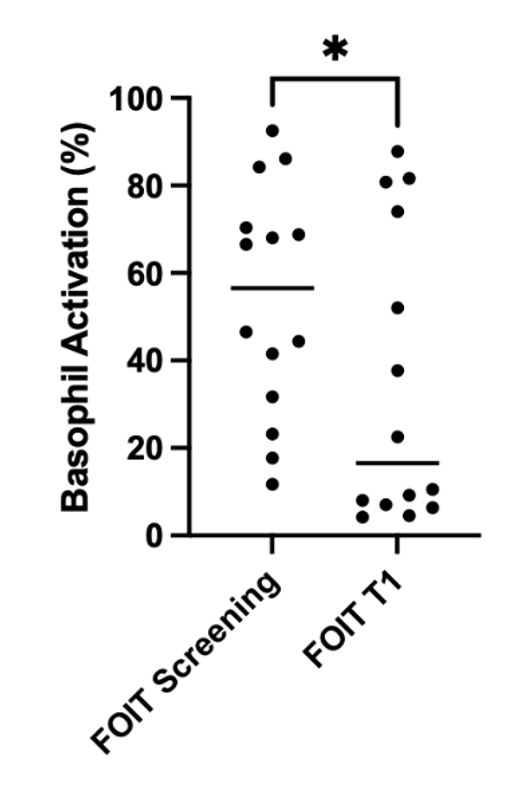

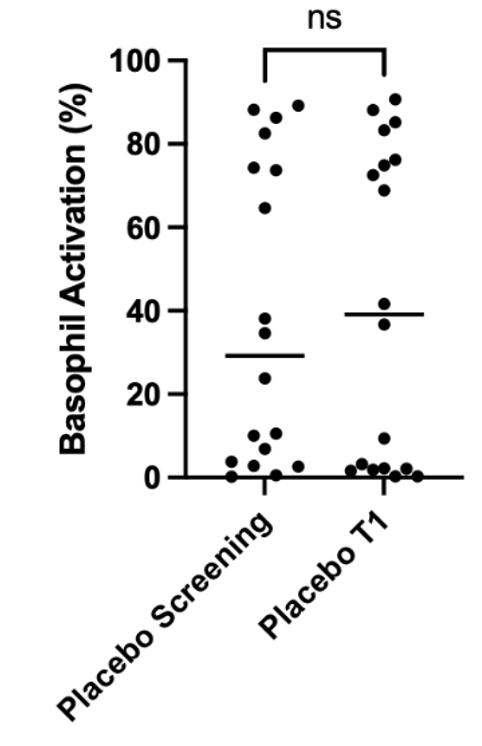


##### Table S11A: Treatment emergent adverse events (TEAEs) by participant, categorized by causality

| **Category**​ | **FOIT (n=35)**​ n (%)​ | **Placebo (n=35)**​ n (%)​ |
| --- | --- | --- |
| **Subjects with TEAEs​** | **35 (100%)​** | **35 (100%)​** |
| Subjects with TEAEs​ judged ***probably or possibly*** related to study treatment * | 28 (80%)​ | 15 (43%)​ |
| Subjects with TEAEs judged ***unlikely to be related*** to study treatment ^#^ | 16 (46%) | 6 (17%) |
| Subjects with eczema flare **^‡^** | 10 (29%) | 4 (11%) |
| Subjects with asthma exacerbation | 0 (0%) | 1 (3%) |
| Subjects with chronic urticaria | 1 (3%) | 0 (0%) |
| Subjects with persistent abdominal pain | 1 (3%) | 0 (0%) |
| Subjects with TEAEs judged ***unrelated*** to treatment ^†^ | 30 (86%) | 33 (94%) |
| Subjects with acute infection or illness | 26 (74%) | 30 (86%) |
| Subjects with accidental allergen exposure-related TEAEs ^§^ | 20 (57%) | 23 (66%) |
| *Episodes of accidental exposure to fish* | *13* | *19* |
| *Episodes of accidental exposure to other food allergens* | *17* | *19* |
| *Episodes of accidental exposure to unknown food allergens* | *7* | *8* |
| *Episodes of accidental exposure to non-food allergens* | *11* | *0* |
| Subjects with TEAEs ***related to DBPCFCs*** | 29 (83%) | 30 (86%) |
| Subjects with T1 DBPCFC-related AEs | 27 (77%) | 30 (86%) |
| Subjects with T2 DBPCFC-related AEs | 12 (34%) | 1 (3%) |
| Abbreviations: TEAE=treatment emergent adverse event; FOIT=Fish oral immunotherapy; DBPCFC=double blind placebo-controlled food challenge  * “Treatment-related TEAEs” include objective signs of IgE mediated allergic reactions happening within 4 hours after taking treatment dose, which cannot be reasonably explained by the subject's clinical state, environment, toxic factors or other treatments.  # “TEAEs unlikely to be related to treatment” include allergic symptoms occurring more than 4 hours after taking treatment dose, or symptoms that can be reasonably explained by the subject's clinical state, environment, toxic factors or other treatments, while symptoms do not follow a known pattern of response for the study treatment.  † “TEAEs unrelated to treatment” include any adverse events where an alternate cause is identified, or that does not meet the criteria for the other categories above.  ‡ Eczema flare includes self-reported worsening of eczema condition or increase in rash relative to subject’s baseline before treatment.  § A total of 48 episodes occurred in 20 participants (57%) in the fish OIT group and a total of 46 episodes occurred in 23 participants (66%) in the placebo group. | | |

##### Table S11B: Treatment emergent adverse events (TEAEs) by participant, categorized by severity *

| **Category**​ | **FOIT (n=35)**​ n (%)​ | **Placebo (n=35)**​ n (%)​ |
| --- | --- | --- |
| **Subjects with mild TEAEs​** | **35 (100%)​** | **35 (100%)​** |
| Subjects with treatment-related mild TEAEs | 28 (80%) | 15 (43%) |
| **Subject with moderate TEAEs** | **11 (31%)** | **10 (29%)** |
| Subjects with treatment-related moderate TEAEs​ | 3 (9%)​ | 0 (0%)​ |
| **Subjects with severe TEAEs​** | **3 (9%)​** | **6 (17%)​** |
| Subjects with treatment-related severe TEAEs​ | 1 (3%)​ | 0 (0%)​ |
| Subjects with severe TEAEs related to accidental exposure to fish | 0 (0%) | 1 (3%) |
| Subjects with severe TEAEs related to accidental exposure to other food allergens | 1 (3%) | 0 (0%) |
| Subjects with non-allergic severe TEAEs | 3 (9%) | 5 (14%) |
| **Subjects with TEAEs requiring hospitalisation (SAEs)** | **4 (11%)​** | **7 (20%)​** |
| Subjects with treatment-related SAEs​ | 0 (0%)​ | 0 (0%)​ |
| Subjects with SAEs related to accidental exposure to other food allergens | 0 (0%)​ | 1 (3%)​ |
| Subjects with SAEs related to acute infection or illness | 4 (11%) | 7 (20%) |
| **Subjects with TEAEs requiring epinephrine​** | **1 (3%)​** | **1 (3%)​** |
| Subjects with treatment-related TEAEs requiring epinephrine​ | 1 (3%)​ | 0 (0%)​ |
| Subjects with SAEs related to accidental exposure to other food allergens requiring epinephrine | 1 (3%)​ | 0 (0%)​ |
| Subject with T1 DBPCFC-related TEAEs requiring epinephrine | 0 (0%) | 1 (3%) |
| * Categorisation of severity of TEAEs are based on ICH guidelines for non-allergic reactions (mild, moderate, severe), and the NIH NIAID CoFAR specific grading system for allergic reactions (Grade 1 – Mild, Grade 2 – Moderate, Grade 3 – Severe, Grade 4 – Life-threatening, Grade 5 – Death).  Note: An SAE is defined as any adverse event that: (1) results in death; or (2) is immediately life threatening; or (3) requires inpatient hospitalization; or (4) required prolongation of existing hospitalization; or (5) resuls in persistent or significant disability/ incapacity; or (6) is a congenital anomaly/ birth defect.​ | | |

##### Table S12: Treatment emergent adverse events during the intervention period

| ​ | **Rush phase**​ | | **Build-up phase**​ | | **Maintenance phase**​ | | **Overall**​ | |
| --- | --- | --- | --- | --- | --- | --- | --- | --- |
| ​ | **FOIT**​  **(n=35)**​ | **Placebo  (n=35)**​ | **FOIT**​  **(n=35)**​ | **Placebo  (n=35)**​ | **FOIT**​  **(n=35)**​ | **Placebo  (n=35)**​ | **FOIT**​  **(n=35)**​ | **Placebo (n=35)**​ |
| **At least one dosing reaction**​ | **12 (34%)*** | **0*** | **23 (66%)** | **14 (40%)** | **11 (31%)** | **5 (14%)** | **28 (80%)§** | **15 (43%)§** |
| At least one dosing reaction ​ requiring epinephrine​ | 0 | 0 | 1 (3%) | 0 | 0 | 0 | 1 (3%) | 0 |
| At least one mild dosing reaction​ | 11 (31%)* | 0* | 23 (66%) | 14 (40%) | 10 (29%) | 5 (14%) | 28 (80%)§ | 15 (43%)§ |
| At least one moderate dosing reaction​ | 1 (3%) | 0 | 1 (3%) | 0 | 1 (3%) | 0 | 3 (9%) | 0 |
| At least one severe dosing reaction ​ | 0 | 0 | 1 (3%) | 0 | 0 | 0 | 1 (3%) | 0 |
| ​ |  |  |  |  |  |  |  |  |
| System organ class and dosing reactions |  |  |  |  |  |  |  |  |
| Cutaneous symptoms​ | 10 (29%)* | 0* | 19 (54%) | 11 (31%) | 7 (20%) | 4 (11%) | 26 (74%)§ | 13 (37%)§ |
| Urticaria​ | 10 (29%)* | 0* | 15 (43%) | 11 (31%) | 6 (17%) | 2 (6%) | 23 (66%)§ | 11 (31%)§ |
| Cutaneous angioedema​ | 2 (6%) | 0 | 10 (29%)^†^ | 0^†^ | 3 (9%) | 1 (3%) | 11 (31%)§ | 1 (3%)§ |
| Gastrointestinal symptoms​ | 1 (3%) | 0 | 5 (14%) | 5 (14%) | 4 (11%) | 1 (3%) | 7 (20%) | 6 (17%) |
| Vomiting​ | 0 | 0 | 2 (6%) | 1 (3%) | 3 (9%) | 0 | 3 (9%) | 1 (3%) |
| Diarrhea​ | 1 (3%) | 0 | 2 (6%) | 3 (9%) | 0 | 0 | 2 (6%) | 3 (9%) |
| Abdominal pain​ | 0 | 0 | 4 (11%) | 1 (3%) | 1 (3%) | 1 (3%) | 5 (14%) | 2 (6%) |
| Respiratory symptoms​ | 1 (3%) | 0 | 8 (23%) | 2 (6%) | 1 (3%) | 3 (9%) | 8 (23%) | 5 (14%) |
| Rhinorrhea/ sneezing​ | 0 | 0 | 3 (9%) | 2 (6%) | 0 | 3 (9%) | 3 (9%) | 5 (14%) |
| Cough​ | 0 | 0 | 3 (9%) | 2 (6%) | 0 | 0 | 3 (9%) | 2 (6%) |
| Hoarse voice​ | 0 | 0 | 2 (6%) | 0 | 0 | 0 | 2 (6%) | 0 |
| Dyspnea | 0 | 0 | 2 (6%) | 0 | 1 (3%) | 0 | 2 (6%) | 0 |
| Throat tightness/ pain​ | 1 (3%) | 0 | 4 (11%) | 1 (3%) | 0 | 0 | 4 (11%) | 1 (3%) |

*Significant difference between 2 groups in the Rush phase with use of Fisher’s exact test.​

† Significant difference between 2 groups in the Build-up phase with use of Fisher’s exact test.​

§ Significant difference between 2 groups overall with use of Fisher’s exact test.​

##### Table S13A: Overall Treatment Adherence Data (N=70 ITT Analysis)

|  | **FOIT**  **(N=35)** | **Placebo**  **(N=35)** |
| --- | --- | --- |
| Subject passed rush induction (Dose 1-6), n (%) ^a^ | 31 (89) | 35 (100) |
| Number of days of FOIT/placebo treatment, median (IQR) | 368 (362, 371) | 367 (362, 371) |
|  |  |  |
| **Subjects completing buildup phase, n (%) ^a^** | **32 (91)** | **35 (100)** |
| Number of days of buildup phase, median (IQR) | 83 (76, 98) | 78 (73, 88) |
|  |  |  |
| Subjects with at least one episode of 5 or more consecutive missed doses during buildup phase, n (%) ^a^ | 10 (29) | 11 (31) |
| Number of episodes of 5 or more consecutive missed doses during buildup phase | 25 | 12 |
| Reasons for missing 5 or more doses during buildup phase, n (%) |  |  |
| Concomitant illness (for example, fever, URTI or other viral infection, gastroenteritis) | 15 (60) | 6 (50) |
| Allergic reaction to dose at home | 1 (4) | 0 (0) |
| Other atopic conditions (for example, eczema, allergic rhinitis, asthma, allergic reaction to other allergens) | 2 (8) | 0 (0) |
| Carer not available for dose administration | 1 (4) | 0 (0) |
| Travel | 6 (24) | 6 (50) |
| % Missed doses during buildup phase |  |  |
| Mean (SD) | 10.2 (11.1) | 7.8 (7.9) |
| Median (IQR) | 7.5 (1.5, 14.0) | 6.0 (0.0, 13.5) |
|  |  |  |
| **Number of episodes of dose adjustment during buildup phase** ^c^ | **8** | **5** |
| Reason for dose adjustment, n (%) |  |  |
| Failed updose with allergic reaction | 2 (25) | 2 (40) |
| Allergic symptoms within 3 days prior to scheduled updose | 2 (25) | 0 (0) |
| Moderate-to-severe treatment-related TEAEs requiring dose reduction ^d^ | 2 (25) | 0 (0) |
| Missing dose for ≥14 days due to travelling | 1 (13) | 2 (40) |
| Missing dose for ≥14 days due to non-allergic illnesses | 1 (13) | 1 (20) |
|  |  |  |
| **Subjects completing maintenance phase, n (%) ^a^** | **30 (86)** | **33 (94)** |
| Number of days of maintenance phase, median (IQR) | 281 (259, 293) | 287 (263, 296) |
|  |  |  |
| Subjects with at least one episode of 5 or more consecutive missed doses during maintenance phase, n (%) ^b^ | 13 (41) | 15 (43) |
| Number of episodes of 5 or more consecutive missed doses during maintenance phase | 18 | 22 |
| Reasons for missing 5 or more doses during maintenance phase, n (%) |  |  |
| Concomitant illness (for example, fever, URTI or other viral infection, gastroenteritis) | 3 (17) | 8 (36) |
| Allergic reaction to dose at home | 0 (0) | 0 (0) |
| Other atopic conditions (for example, eczema, allergic rhinitis, asthma, allergic reaction to other allergens) | 1 (6) | 0 (0) |
| Travel | 13 (72) | 9 (41) |
| Subject refuse doses | 1 (6) | 4 (18) |
| Festival celebration | 0 (0) | 1 (5) |
| % Missed doses during maintenance phase |  |  |
| Mean (SD) | 11.0 (11.4) | 9.9 (11.1) |
| Median (IQR) | 6.5 (4.0, 16.0) | 6.0 (1.0, 16.0) |
|  |  |  |
| Number of episodes of dose adjustment during maintenance phase | 5 | 2 |
| Reason for dose adjustment, n (%) |  |  |
| Missing dose for ≥14 days due to travelling | 2 (40) | 1 (50) |
| Missing dose for ≥14 days due to non-allergic illnesses | 2 (40) | 0 (0) |
| Missing dose for ≥14 days due to non-compliance | 1 (20) | 1 (50) |
|  |  |  |
| ^a^ Percentage calculation with denominator as number of subjects randomized  ^b^ Percentage calculation with denominator as number of subjects entering maintenance phase (= number of subjects completing buildup phase) | | |

^c^ Dose modifications were performed according to pre-specified protocol criteria (see full protocol)

^d^ One 4-year-old FOIT participant experienced persistent abdominal pain and occasional vomiting requiring dose reduction during treatment. EoE was considered but deemed less likely given the absence of typical EoE features including feeding difficulties with other foods, dysphagia, food impaction, or failure to thrive. Endoscopy was declined, precluding histologic confirmation. This participant subsequently withdrew from the study.

##### Table S13B: Comparison of Participants by Adherence: Those Missing ≥5 Doses vs <5 Doses

|  | **Miss 5 or more doses**  **(n = 41)** | **Miss less than 5 doses**  **(n = 29)** | **P value** |
| --- | --- | --- | --- |
| DS, n (%) | 11 (27) | 8 (28) | 1.000 |
| SU, n (%) | 6 (15) | 5 (17) | 1.000 |
| Months of age, median (IQR) | 62 (40, 79) | 76 (38, 98) | 0.295 |
| **Baseline, median (IQR)** | | | |
| Total IgE | 909 (426, 2042) | 860 (338, 1871) | 0.230 |
| sIgE (codfish) | 3.2 (1.4, 11.4) | 3.4 (0.9, 7.0) | 0.453 |
| sIgE (rGad c1) | 12.8 (4.5, 41.3) | 10.0 (2.3, 25.0) | 0.180 |
| sIgG4 (codfish) | 0.6 (0.2, 1.5) | 0.5 (0.1, 1.9) | 0.499 |
| SPT (codfish) | 4.0 (3.5, 5.5) | 4.5 (3.0, 6.5) | 0.688 |
| BAT (codfish) | 73.1 (22.8, 83.9) | 63.1 (4.3, 81.1) | 0.362 |
| **T1, median (IQR)** | | | |
| Total IgE | 789 (459, 2359) | 604 (326, 1707) | 0.391 |
| sIgE (codfish) | 1.8 (1.1, 5.5) | 1.7 (1.0, 4.9) | 0.512 |
| sIgE (rGad c1) | 6.7 (3.1, 32.0) | 4.8 (3.1, 15.5) | 0.537 |
| sIgG4 (codfish) | 1.0 (0.3, 3.9) | 0.9 (0.3, 2.3) | 0.507 |
| SPT (codfish) | 3.3 (2.5, 5.4) | 3.5 (2.5, 5.5) | 0.997 |
| BAT (codfish) | 55.5 (37.0, 71.6) | 83.6 (29.8, 95.4) | 0.313 |

##### Table S14 – Treatment adherence data during build-up phase

| ​ | **FOIT (N=35)**​ | **Placebo** **(N=35)**​ |
| --- | --- | --- |
| Subject completing rush induction (Dose 1-6), n (%) a​ | 31 (89)​ | 35 (100)​ |
| Time of treatment, median days (IQR)​ | 368 (362, 371)​ | 367 (362, 371)​ |
| Total time of treatment, patient-years b​ | 31.70​ | 33.96​ |
| **Build-up Phase**​ | ​ | ​ |
| **Subjects completing buildup phase, n (%) c**​ | **32 (91)**​ | **35 (100)**​ |
| Number of treatment days, median (IQR)​ | 81 (75, 92)​ | 78 (73, 88)​ |
| Subject with absent treatment diary, n (%) c​ | 1 (3)​* | 1 (3)​ |
| Subject with incomplete treatment diary, n (%) c​ | 2 (6)​ | 2 (6)​ |
| ​ | ​ | ​ |
| **Dose adherence rate**​ | ​ | ​ |
| Median % (IQR) d​ | 93.5 (86, 99)​ | 93.5 (86, 100)​ |
| Mean % (SD) d​ | 90.3 (11.0)​ | 91.3 (9.67)​ |
|  | ​ | ​ |
| Subjects with at least one episode of 5 or more consecutive missed doses, n (%) c​ | 10 (29)​ | 11 (31)​ |
| Total number of episodes of 5 or more consecutive missed doses​ | 25​ | 12​ |
| Reasons for missing 5 or more doses, n (%) e​ | ​ | ​ |
| Concomitant illness/ viral infection​ | 15 (60)​ | 6 (50)​ |
| Allergic reaction to dose at home​ | 1 (4)​ | 0 (0)​ |
| Other atopic conditions​ | 2 (8)​ | 0 (0)​ |
| Carer not available for dose administration​ | 1 (4)​ | 0 (0)​ |
| Travel​ | 6 (24)​ | 6 (50)​ |
| * 2 participants with missing diary withdrawn during build-up phase  [a] Subjects who reacted to any dose and therefore did not complete the rush induction started the buildup phase at the dose immediately below the reaction-eliciting dose; the remaining rush doses that were not completed were be incorporated into the buildup phase with an extended buildup schedule.​  [b] Total treatment time in patient-years was calculated by the sum of days of treatment of subjects in the FOIT/Placebo group divided by 365.25​  [c] Percentage was calculated using a denominator as number of subjects entering build-up phase (FOIT=35; Placebo=35)​  [d] Dose adherence rate was calculated as the total number of doses administered divided by the number of treatment days during the build‑up phase; subjects with no build‑up diary were excluded; for subjects who returned only part of the build‑up diary, the rate was estimated from the available build-up records. ​  [e] Percentage was calculated using a denominator as total number of episodes of 5 or more consecutive missed doses in the phase.​ | | |

##### Table S15 – Treatment adherence data during maintenance phase

| ​ | **FOIT(N=35)**​ | **Placebo** **(N=35)**​ |
| --- | --- | --- |
| **Maintenance Phase**​ | ​ | ​ |
| **Subjects completing maintenance phase, n (%) c**​ | **30 (86)**​ | **33 (94)**​ |
| Number of treatment days, median (IQR)​ | 281 (259, 293)​ | 287 (263, 296)​ |
| ​ | ​ | ​ |
| Subject with absent treatment diary, n (%) f​ | 3 (9)​* | 4 (11)*​ |
| Subject with incomplete treatment diary, n (%) f​ | 4 (13)​ | 10 (29)​ |
| ​ | ​ | ​ |
| **Dose adherence rate**​ | ​ | ​ |
| Median % (IQR) ^g​^ | 93.5 (83.5, 97)​ | 93 (84, 99)​ |
| Mean % (SD) g​ | 88.8 (11.5)​ | 87.8 (15.50)​ |
| ​ | ​ | ​ |
| Subjects with at least one episode of 5 or more consecutive missed doses, n (%) d​ | 13 (41)​ | 15 (43)​ |
| Total number of episodes of 5 or more consecutive missed doses​ | 18​ | 22​ |
| Reasons for missing 5 or more doses, n (%) e​ | ​ | ​ |
| Concomitant illness/ viral infection​ | 3 (17)​ | 8 (36)​ |
| Allergic reaction to dose at home​ | 0 (0)​ | 0 (0)​ |
| Other atopic conditions​ | 1 (6)​ | 0 (0)​ |
| Travel​ | 13 (72)​ | 9 (41)​ |
| Subject refuse doses​ | 1 (6)​ | 4 (18)​ |
| Festival celebration​ | 0 (0)​ | 1 (5)​ |
| * 4/7 participants with missing diary withdrawn during maintenance phase  [c] Percentage was calculated using a denominator as number of subjects entering build-up phase (FOIT=35; Placebo=35)​  [d] Dose adherence rate was calculated as the total number of doses administered divided by the number of treatment days during the build‑up phase; subjects with no build‑up diary were excluded; for subjects who returned only part of the build‑up diary, the rate was estimated from the available build-up records. ​  [e] Percentage was calculated using a denominator as total number of episodes of 5 or more consecutive missed doses in the phase.​  [f] Percentage was calculated using a denominator as number of subjects entering maintenance phase (FOIT=32; Placebo=35)​  [g] Dose adherence rate was calculated as the total number of doses administered divided by the number of treatment days during the maintenance phase; for subjects whose maintenance diary was not returned, the rate was estimated from the build‑up diary record; for subjects whose maintenance diary was only partially returned, the rate was estimated from the available maintenance records; subjects who returned neither a build‑up nor a maintenance diary were excluded.​ | | |

##### Table S16 – Withdrawal Rates and Reasons by Study Phase

|  | **FOIT** | **Placebo** |
| --- | --- | --- |
|  | n=35 | n=35 |
| Withdrawal rates, n (%) | 5 (14.3) | 2 (5.7) |
| Withdrawal reasons |  |  |
| Buildup phase, n (%) | 3 (8.6) | 0 (0) |
| Uncontrolled eczema | 1 | 0 |
| Adverse events | 1 | 0 |
| Emigration | 1 | 0 |
| Maintenance phase, n (%) | 2 (5.7) | 2 (5.7) |
| Uncontrolled eczema | 1 | 0 |
| Non-compliance | 1 | 1 |
| Emigration | 0 | 1 |

##### Figure S7: Codfish-specific antibody levels and IgG4/IgE ratios during the avoidance period.

(A) Codfish-specific IgE (sIgE) levels at baseline, end of treatment (T1), and after 8-week avoidance (T2) in the sustained unresponsiveness (SU, blue) and desensitization (DS, green) groups. **(B)** Codfish-specific IgG4 (sIgG4) levels at baseline, T1 and T2 in both groups. A decrease in sIgG4 levels was observed in the DS group following the avoidance period, while levels remained stable in the SU group. **(C)** Codfish sIgG4/sIgE ratio at baseline, T1 and T2 in both groups. Data are presented as individual values with median. P values derived from Friedman test (across 3 timepoints) and Wilcoxon signed-rank test (pairwise, Bonferroni-adjusted). *P < 0.05, **P < 0.01, ***P < 0.001. SU, sustained unresponsiveness group; DS, desensitization group; T1, baseline; T2, after 8-week avoidance.

**
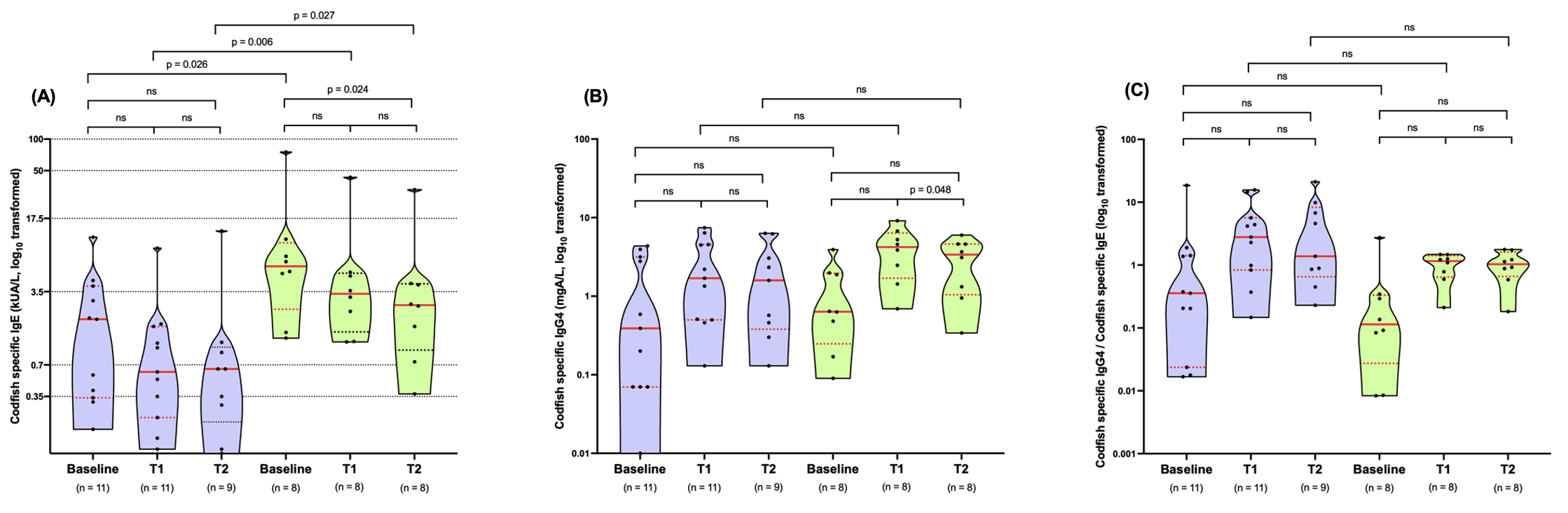
**

##### Figures S8: Methylmercury level in the red blood cells of codfish OIT vs placebo participants measured at week 52. Levels below 1 ng/ml are often considered normal for this age.

 
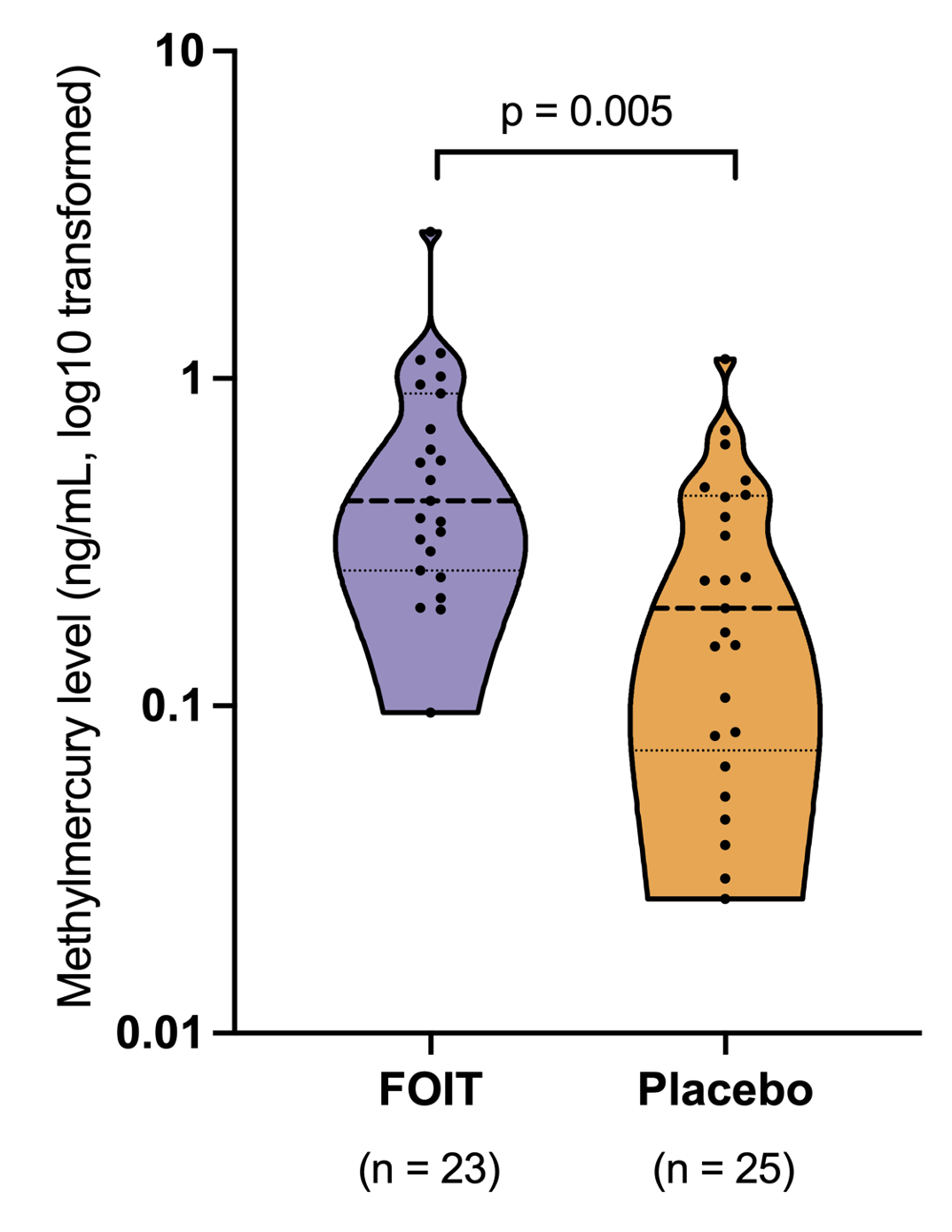


##### Figure S9: Median (IQR) SCORAD scores of codfish OIT vs placebo participants during the treatment phase.

**
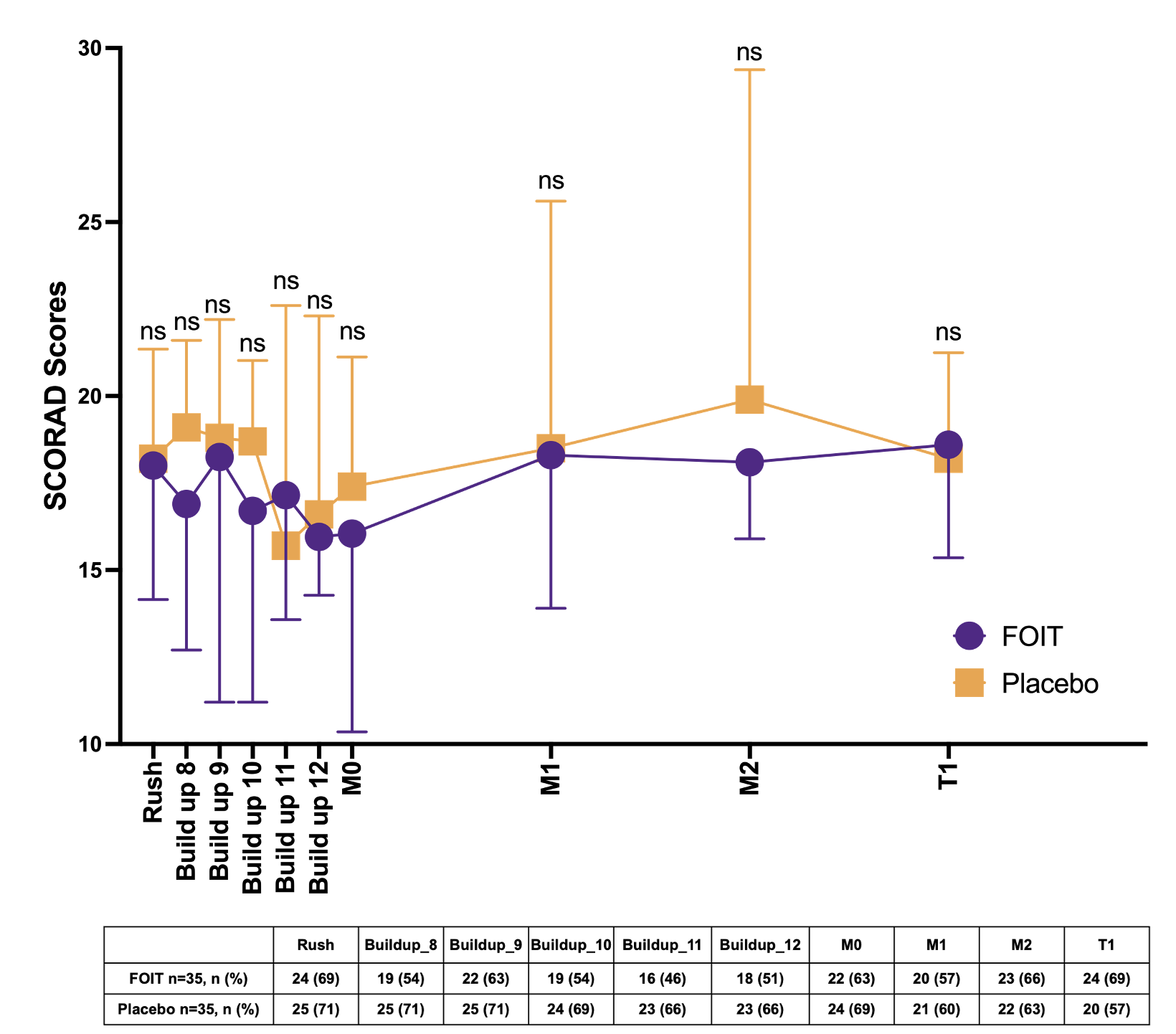
**

##### Figure S10: Urinary microplastic levels in study participants.

Microplastic concentrations detected in urine samples from a subset of participants at T1 (52 weeks of treatment). **(A)** Comparison of total urinary microplastic levels between fish oral immunotherapy (FOIT) treatment and placebo groups. **(B)** Total microplastic levels and polypropylene-specific microplastic levels stratified by treatment outcome (sustained unresponsiveness [SU], desensitization [DS], and persistent allergy [PA] groups). Microplastics were identified using spectroscopic matching indices at 70% (HQI70, standard threshold) thresholds. Data are presented as individual values with median. No significant differences in urinary microplastic levels were observed between treatment groups or outcome groups. Statistical comparisons were performed using **Mann-Whitney U test**. NS, not significant; HQI, high quality index; FOIT, fish oral immunotherapy; SU, sustained unresponsiveness; DS, desensitization; PA, persistent allergy; T1, baseline (52 weeks of treatment).


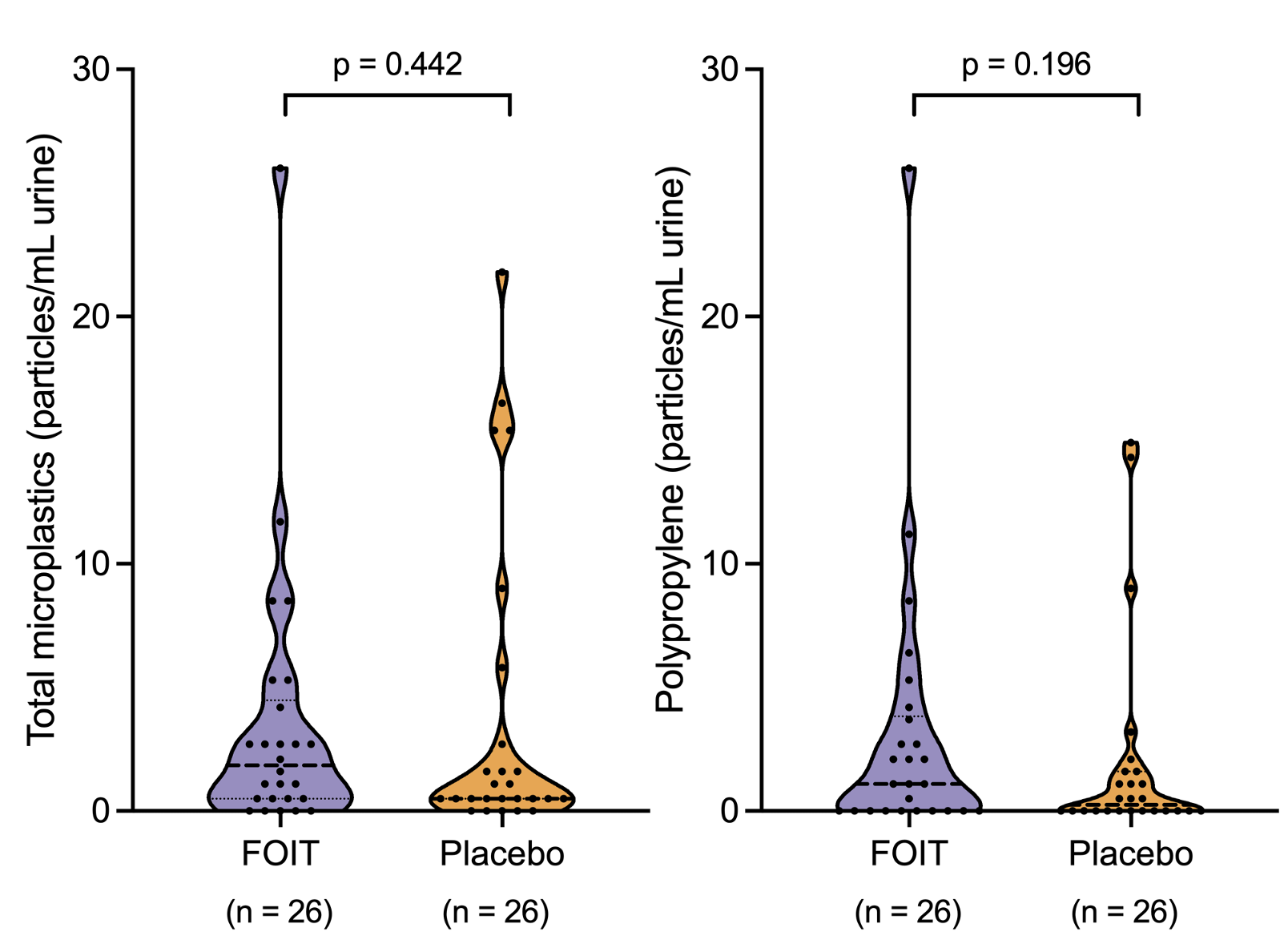


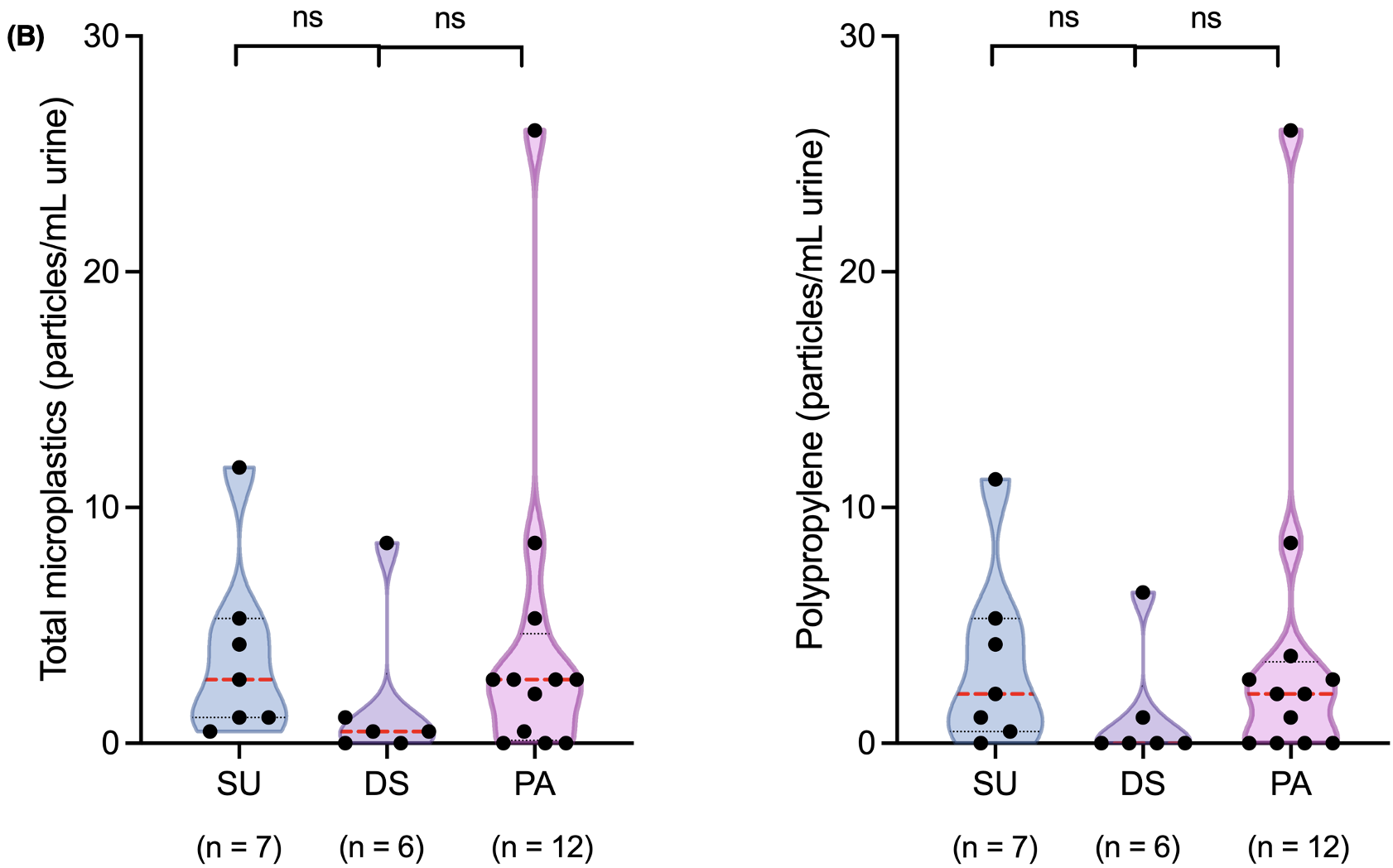

Supplement: Supplementary file 1 — Data S1: all70268‐sup‐0001‐TableS1‐S16‐FigureS1‐S10.docx. [file ALL-81-1799-s001.docx]
